# Identification of Novel AChE-Targeting Neuroprotective Peptides from Pacific Oyster (*Crassostrea gigas*): An Integrated Pipeline of Peptidomics, Molecular Dynamics, and Cellular Validation

**DOI:** 10.3390/md24090298

**Published:** 2026-08-25

**Authors:** Shi-Kun Suo, Kuo Dang, Ying-Ying Zhang, Yao-Yao Zhang, Yu-Xin Luo, Jun-Wei Yan, Dao-Dong Pan, Yan-Li Wang, Long Li, Chao-Ying Zhang, Xin-Chang Gao, Ya-Li Dang

**Affiliations:** 1 College of Food Science and Engineering, Ningbo University, Ningbo 315211, China; 2 Institute of Drug Discovery Technology, Ningbo University, Ningbo 315211, China; 3 School of Pharmacy, Health Science Center, Ningbo University, Ningbo 315211, China; 4 Engineering Research Center of Storage and Processing of Xinjiang Characteristic Fruits and Vegetables, Ministry of Education, School of Food Science and Technology, Shihezi University, Shihezi 832000, China; 5 Department of Quality and Safety, Chinese Academy of Fishery Sciences, Beijing 100141, China; 6 Qian Xuesen Collaborative Research Center of Astrochemistry and Space Life Sciences, Ningbo University, Ningbo 315211, China

**Keywords:** Pacific oyster (*Crassostrea gigas*), acetylcholinesterase, molecular dynamics simulation, peptides, neuroprotection, in silico screening

## Abstract

Although the Pacific oyster (*Crassostrea gigas*) is a premium marine protein source, its neuroprotective peptidome remains largely uncharacterized. This study established an integrated in silico and in vitro pipeline to discover acetylcholinesterase (AChE)-targeting peptides with cellular AChE-regulating and neuroprotective peptides from simulated gastrointestinal digests of oyster. Peptidomic profiling identified 18,292 sequences, which were filtered down to seven candidates predicted to have favorable blood–brain barrier (BBB) permeability and to be non-toxic and non-allergenic (VPYPR, VPVHF, HHTF, PVHF, GPKPW, HWF, and KYW) via multi-step virtual screening. In cellular assays, simulated H_2_O_2_ injury (500 μM) reduced PC12 cell viability to 47.53 ± 4.53%. Compared with the model group, pretreatment with the three most potent candidates—HHTF, VPYPR, and VPVHF (200 μM)—significantly rescued injured cells, restoring cell viability to 88.31 ± 7.83%, 85.12 ± 3.35%, and 82.00 ± 3.47%, respectively (*p* < 0.05). These peptides effectively fortified cellular antioxidant defenses by increasing glutathione (GSH) levels to 24.24, 30.11, and 26.83 nmol/mg protein (from 20.22 nmol/mg protein in the model group) and superoxide dismutase (SOD) activity to 151.41, 153.97, and 151.96 U/mg protein (from 119.33 U/mg protein), while suppressing malondialdehyde (MDA) accumulation to 0.088, 0.064, and 0.086 nmol/mg protein (from 0.193 nmol/mg protein). Crucially, the peptides alleviated cholinergic dysfunction by normalizing the H_2_O_2_-induced elevation of intracellular AChE activity (11.39 nmol/min/mg protein) down to 7.02, 6.22, and 7.14 nmol/min/mg protein, respectively. Specifically, VPYPR (200 μM) restored AChE activity to a level (6.22 nmol/min/mg protein) that was not significantly different from that in the normal control group (*p* > 0.05). Molecular dynamics (MD) simulations (100 ns) and molecular mechanics Poisson–Boltzmann surface area (MM-PBSA) calculations identified VPYPR as the leading candidate with a remarkably low binding free energy of −49.74 ± 3.58 kcal/mol. This study demonstrates that oyster gastrointestinal digests are valuable reservoirs of multi-target neuroprotective ingredients and provides an efficient strategy for marine bioactive peptide discovery.

## 1. Introduction

The global increase in life expectancy has been accompanied by a rising prevalence of age-related neurodegenerative disorders, particularly Alzheimer’s disease (AD), which is characterized by progressive cognitive decline, memory impairment, and neuronal dysfunction [1]. Although AD pathogenesis is multifactorial, two central pathological contributors are widely recognized: oxidative stress and cholinergic dysfunction [2,3]. Excessive reactive oxygen species (ROS) induce neuronal damage through lipid peroxidation, mitochondrial dysfunction, and apoptosis, while accelerated hydrolysis of acetylcholine by acetylcholinesterase (AChE) leads to impaired cholinergic neurotransmission and cognitive deficits [4]. Current pharmacological interventions for AD rely primarily on AChE inhibitors such as donepezil, rivastigmine, and galantamine [5]. However, their clinical benefits are often limited by adverse effects including gastrointestinal discomfort, hepatotoxicity, and poor long-term efficacy [6]. Consequently, increasing attention has turned toward safer dietary strategies and food-derived bioactive compounds capable of modulating oxidative injury and cholinergic imbalance.

Bioactive peptides generated from food proteins have emerged as attractive functional ingredients owing to their high biocompatibility, structural diversity, and multiple physiological activities [7]. Depending on amino acid composition and sequence characteristics, these peptides may exert antioxidant, anti-inflammatory, antihypertensive, and neuroprotective effects [8,9]. Marine organisms, in particular, are considered valuable reservoirs of functional peptides due to their unique biodiversity and protein composition. Pacific oyster (*Crassostrea gigas*), one of the most commercially important shellfish species worldwide, is rich in high-quality proteins and balanced essential amino acids [10]. However, previous studies on Pacific oyster have mainly focused on nutritional value and general biological activities, leaving the neuroprotective potential of peptides released during gastrointestinal digestion largely unexplored.

Traditional peptide discovery methods rely heavily on activity-guided fractionation and trial-and-error experimental screening, which are time-consuming, resource-intensive, and inherently low-throughput. Moreover, the complex nature of gastrointestinal digests, often containing thousands of peptide sequences, poses a significant challenge for conventional approaches [11]. In this context, computational methods offer a powerful alternative. Given its critical role in terminating synaptic neurotransmission and its clinical validation in neurodegenerative disorders, AChE was selected as the core target to evaluate the inhibitory potency and molecular binding mechanisms of the identified peptides. Molecular docking enables rapid prediction of binding affinities between peptides and target proteins such as AChE, while molecular dynamics (MD) simulations provide atomistic insight into the stability, flexibility, and interaction patterns of peptide–protein complexes under near-physiological conditions. MM-PBSA calculations further quantify binding free energies and decompose energetic contributions by residue, allowing direct comparison and ranking of candidate peptides. These in silico tools, when integrated with peptidomics and focused experimental validation, can substantially accelerate the discovery of bioactive peptides from complex food matrices.

In the present study, we developed an integrated computational–experimental pipeline to identify AChE-targeting neuroprotective peptides from simulated gastrointestinal digests of Pacific oyster. The workflow combines simulated gastrointestinal digestion and peptidomic sequencing, multi-step in silico screening covering bioactivity, safety, and blood–brain barrier permeability, molecular docking against AChE, 100 ns molecular dynamics simulations to assess binding stability, MM-PBSA calculations to quantify binding free energies, and experimental validation in an H_2_O_2_-induced oxidative stress model using PC12 cells. This approach not only identifies promising candidate peptides but also elucidates the molecular basis of their AChE-targeting behavior at an atomistic level.

## 2. Results

### 2.1. Compositional Characteristics of Simulated Gastrointestinal Digests of Pacific Oyster

**Figure 1:**
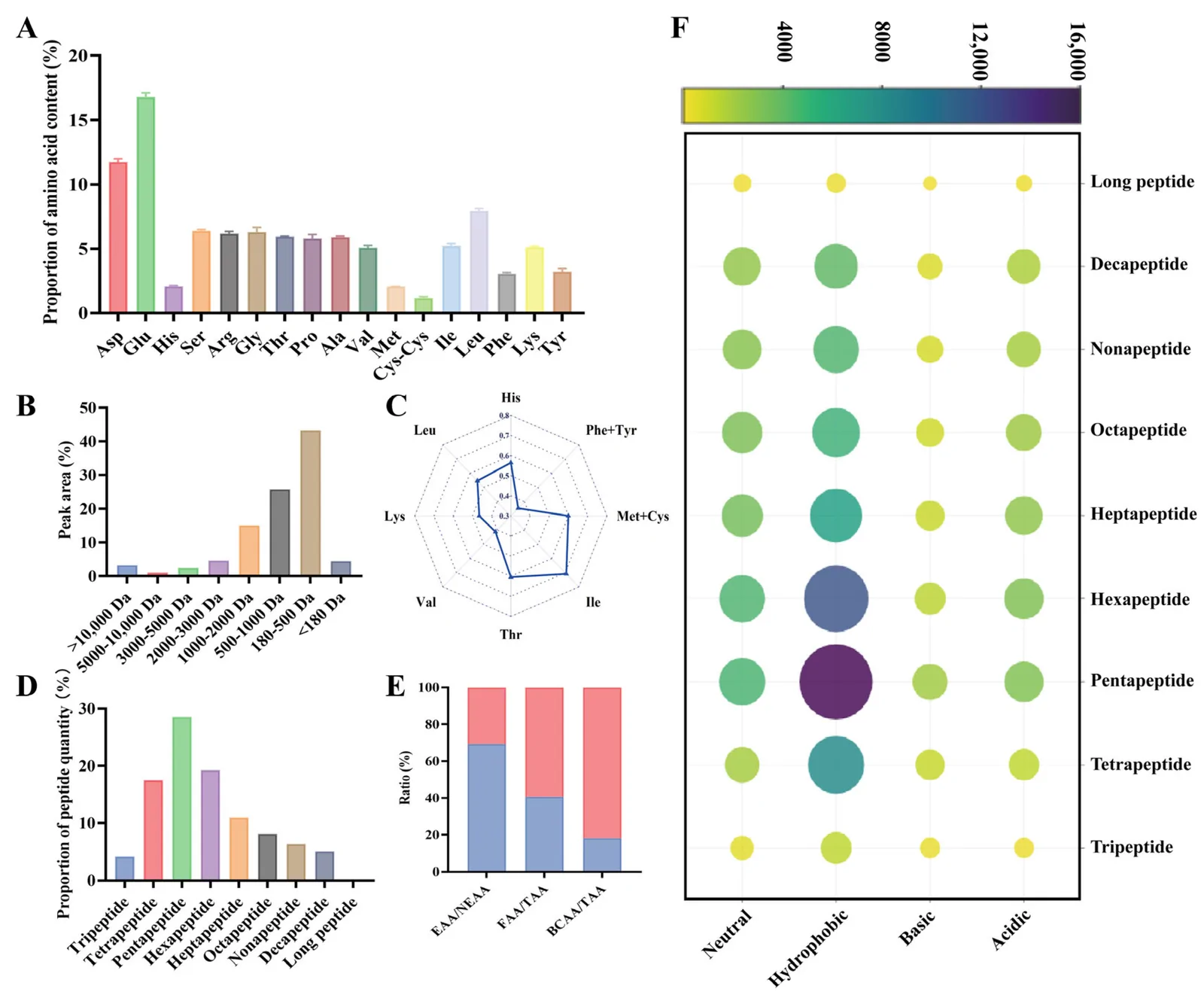
Compositional characteristics of the simulated digesta of Pacific oyster. (**A**) Amino acid composition determined experimentally by means of HPLC after acid hydrolysis. (**B**) Molecular weight distribution. (**C**) Amino acid scores. (**D**) Peptide length distribution. (**E**) Ratios of essential/branched-chain amino acids. (**F**) Distribution of amino acid types in peptides of different lengths. Abbreviations: HPLC, high-performance liquid chromatography; EAA, essential amino acids; NEAA, non-essential amino acids; FAA, free amino acids; TAA, total amino acids; BCAA, branched-chain amino acids.

The simulated gastrointestinal digestion of Pacific oyster proteins was performed to evaluate the release of amino acids and peptide fractions. The total amino acid composition of the resulting digests was experimentally determined via acid hydrolysis and HPLC analysis, as shown in Figure 1A. Glutamic acid was the most abundant amino acid, accounting for 16.78 ± 0.33% of the total amino acids, followed by aspartic acid at 11.74 ± 0.24%. Basic amino acids, including arginine (6.16 ± 0.18%), lysine (5.14 ± 0.07%), and histidine (2.08 ± 0.07%), collectively represented 13.38% of the total pool. Hydrophobic amino acids (Leu, Ile, Val, Ala, Pro, Phe, Met, and Tyr) constituted a substantial proportion of the digests.

Molecular weight (MW) distribution analysis (Figure 1B) revealed extensive proteolysis, with the 180–500 Da fraction representing the largest proportion (43.34%), followed by 500–1000 Da (25.79%) and 1000–2000 Da (15.05%). Peptide fractions below 2000 Da accounted for 84.18% of the total digests. Evaluation of amino acid scores identified isoleucine with the highest score and phenylalanine plus tyrosine as the limiting amino acids (Figure 1C). Peptide length distribution analysis (Figure 1D) showed that pentapeptides predominated (28.53%), followed by hexapeptides (19.26%) and tetrapeptides (17.50%). The ratio of essential amino acids (EAA) to non-essential amino acids (NEAA) was 0.69, while branched-chain amino acids (BCAA) represented 18.24% of total amino acids (TAA) (Figure 1E). The distribution of residue types across various peptide lengths (Figure 1F) indicated that hydrophobic amino acids were highly abundant, particularly within pentapeptides and hexapeptides.

### 2.2. Computational Screening and Virtual Prioritization of AChE-Targeting Peptides

Peptidomic sequencing of simulated gastrointestinal digests yielded 18,292 distinct (unique) peptide primary sequences consistently detected across replicate analyses (encompassing both protein-unique and shared/homologous precursor matches). Applying an abundance-based filter (top 10%) narrowed the candidate pool to 1829 sequences traceable to oyster proteins. Among these, 820 sequences contained basic residues. After excluding peptides with isoelectric points (pI) < 7.4, PeptideRanker screening (score > 0.5) yielded 140 candidate sequences. Subsequent blood–brain barrier (BBB) permeability prediction narrowed the selection to 65 peptides. Safety screening predicted that all 65 candidates were non-toxic and non-allergenic.

Based on molecular docking scores against acetylcholinesterase (AChE) and sequence diversity, seven representative peptides were selected for chemical synthesis and biological validation: VPYPR, VPVHF, HHTF, PVHF, GPKPW, HWF, and KYW (Table 1). Their predicted properties, including pI (7.85–10.85), PeptideRanker scores (0.506–0.990), and docking scores (−10.6 to −11.1 kcal/mol), are compiled in Table 1. VPYPR achieved the lowest binding energy (−11.1 kcal/mol), followed by VPVHF (−10.9 kcal/mol).

### 2.3. Cytoprotective and Antioxidant Effects in H_2_O_2_-Induced PC12 Cells

#### 2.3.1. Establishment of a H_2_O_2_-Induced Oxidative Damage Model in PC12 Cells

**Figure 2:**
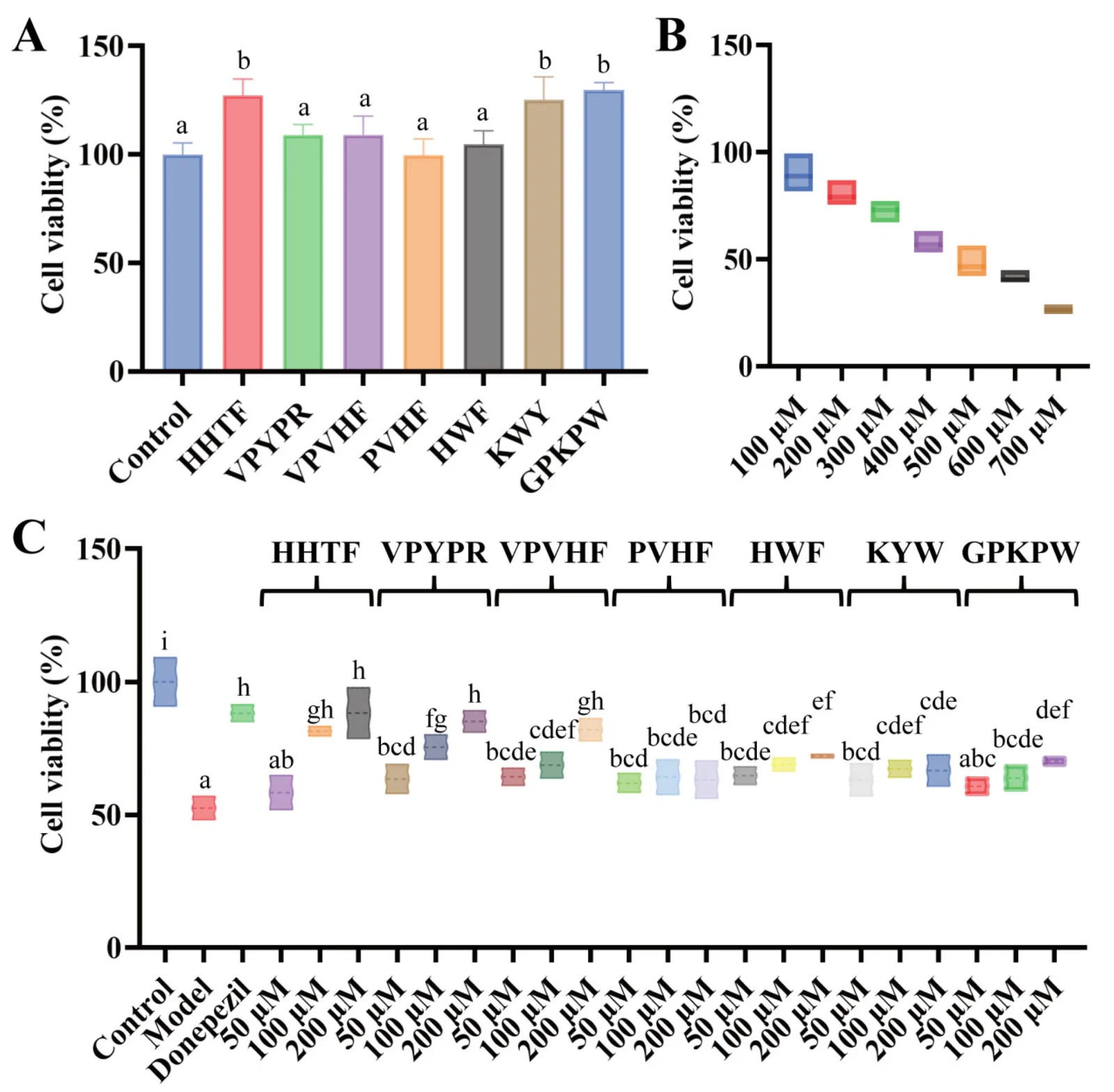
Activity evaluation of the candidate peptides. (**A**) Cytotoxicity of the seven peptides in PC12 cells. (**B**) Establishment of the H_2_O_2_-induced oxidative damage model in PC12 cells. (**C**) Protective effects of the peptides against H_2_O_2_-induced damage. Different letters indicate statistically significant differences between groups (*p* < 0.05).

No significant cytotoxicity was observed in normal PC12 cells treated with the seven synthesized peptides up to 200 μM. Instead, cells treated with HHTF and GPKPW displayed slightly elevated cell viability compared to the control group (Figure 2A). Exposure of PC12 cells to H_2_O_2_ (100–700 μM) for 1 h reduced cell viability in a concentration-dependent manner (Figure 2B). Specifically, treatment with 500 μM H_2_O_2_ decreased cell viability to 47.53 ± 4.53%, which was selected as the optimal injury condition.

Pretreatment with all seven peptides rescued PC12 cells from H_2_O_2_-induced damage in a dose-dependent manner (Figure 2C). The three most effective candidates—HHTF, VPYPR, and VPVHF—at a concentration of 200 μM significantly enhanced cell survival to 88.31%, 85.12%, and 82.00%, respectively, compared to the model group (47.53 ± 4.53%).

#### 2.3.2. Evaluation of the Activity of HHTF, VPYPR, and VPVHF on PC12 Cells

**Figure 3:**
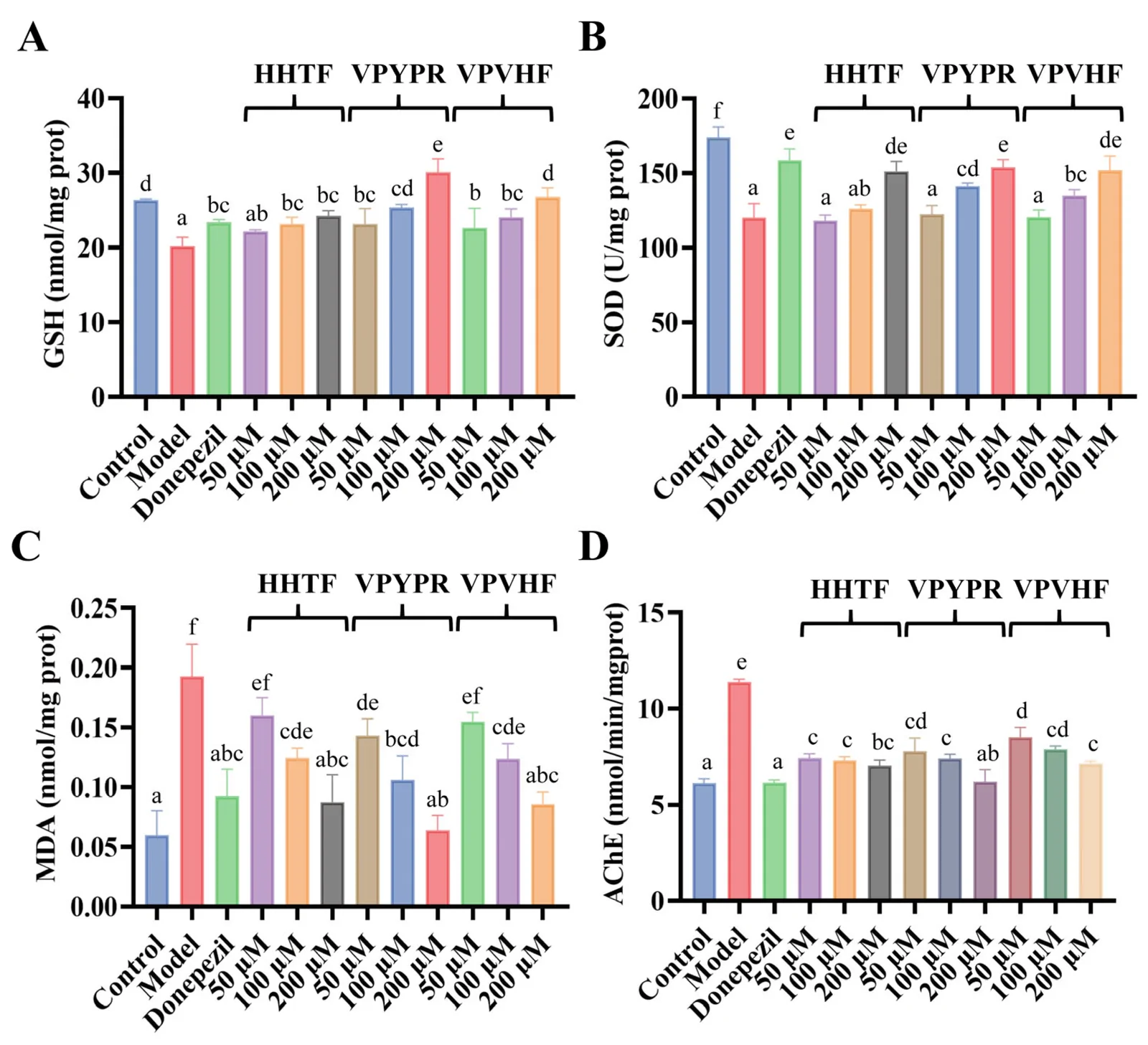
Effects of oyster-derived peptides and donepezil on antioxidant indices and intracellular AChE activity in H_2_O_2_-treated PC12 cells. (**A**) Intracellular GSH level. (**B**) SOD activity. (**C**) MDA content. (**D**) Intracellular AChE activity. Data are expressed as mean ± SD from three independent experiments. Different letters indicate significant differences among groups. Different letters indicate statistically significant differences between groups (*p* < 0.05). Abbreviations: GSH, glutathione; SOD, superoxide dismutase; MDA, malondialdehyde; AChE, acetylcholinesterase; SD, standard deviation.

Mechanism-level evaluation showed that H_2_O_2_ exposure reduced intracellular glutathione (GSH) levels to 20.22 nmol/mg protein and suppressed superoxide dismutase (SOD) activity to 119.33 U/mg protein (Figure 3A,B). Pretreatment with HHTF, VPYPR, and VPVHF (200 μM) restored GSH levels to 24.24, 30.11, and 26.83 nmol/mg protein, respectively, and SOD activity to 151.41, 153.97, and 151.96 U/mg protein, respectively. Concurrently, malondialdehyde (MDA) accumulation, which surged to 0.193 nmol/mg protein in the model group, was significantly suppressed to 0.088 (HHTF), 0.064 (VPYPR), and 0.086 (VPVHF) nmol/mg protein following peptide pretreatment (200 μM, Figure 3C). As a positive control, donepezil significantly increased the GSH level from 20.22 ± 1.21 nmol/mg protein in the model group to 23.39 ± 0.40 nmol/mg protein and significantly reduced MDA accumulation from 0.193 ± 0.027 to 0.092 ± 0.023 nmol/mg protein (*p* < 0.05).

Intracellular AChE activity was markedly elevated in H_2_O_2_-treated PC12 cells, increasing from 6.14 ± 0.23 nmol/min/mg protein in the control group to 11.39 ± 0.16 nmol/min/mg protein in the model group (Figure 3D). Donepezil, used as the positive control, reduced intracellular AChE activity to 6.16 ± 0.15 nmol/min/mg protein, corresponding to a 45.92% reduction relative to the model group. The AChE activity of the donepezil group was not significantly different from that of the normal control group (*p* > 0.05). Pretreatment with HHTF, VPYPR, and VPVHF at 200 μM reduced AChE activity to 7.02, 6.22, and 7.14 nmol/min/mg protein, respectively. Notably, the VPYPR-treated group also showed no significant difference from the normal control group (*p* > 0.05).

### 2.4. Molecular Docking and Interaction Modes with AChE

**Figure 4:**
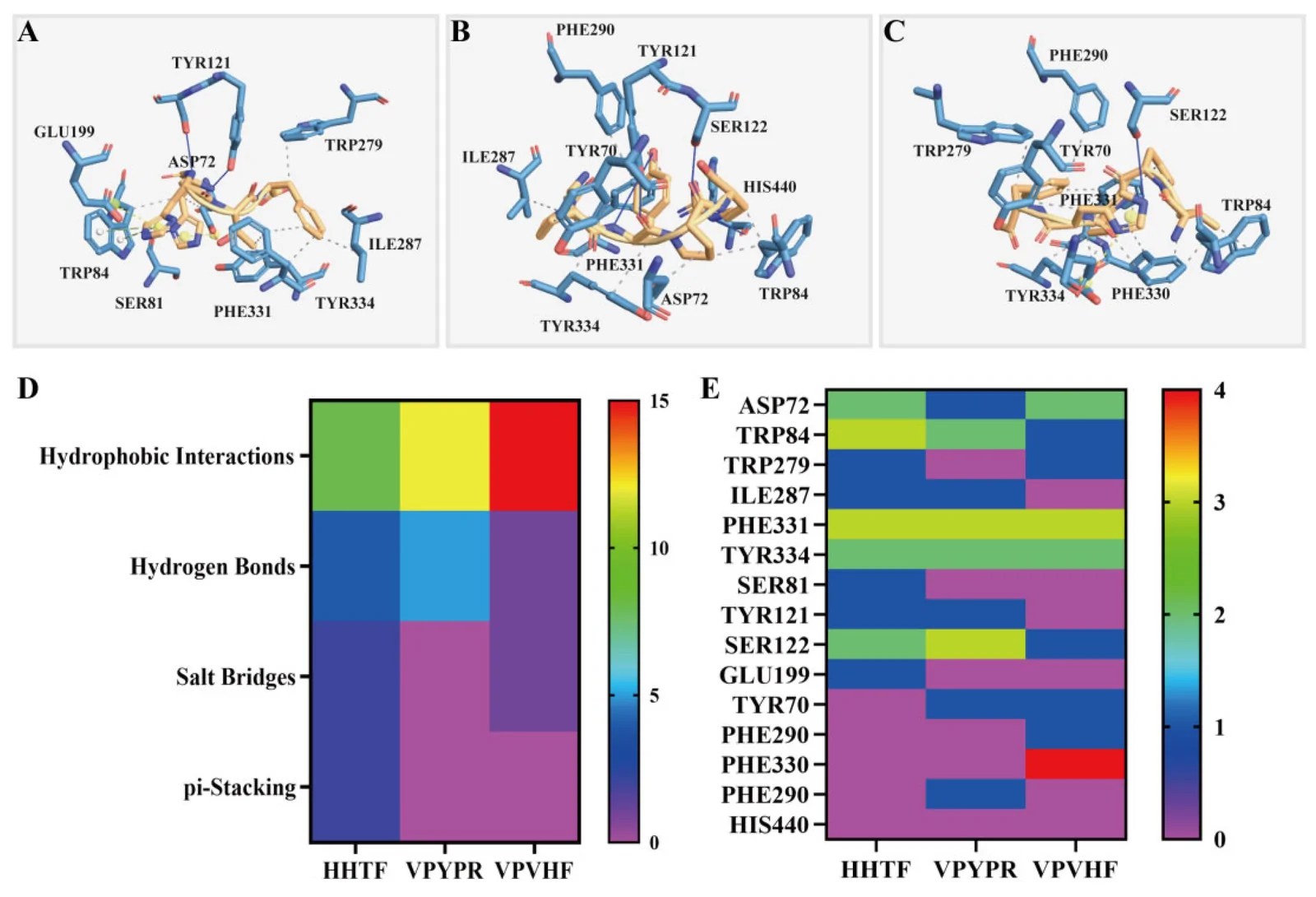
Molecular docking of the three AChE-targeting peptides. (**A**) HHTF. (**B**) VPVHF. (**C**) VPYPR. Blue sticks represent the key interaction residues of then AChE, and yellow sticks represent the ligand peptides. (**D**) Heatmap of interaction forces between peptides and AChE. (**E**) Heatmap of interaction residues. Abbreviation: AChE: acetylcholinesterase.

To elucidate the binding modes of HHTF, VPYPR, and VPVHF, molecular docking was performed against the crystal structure of AChE (PDB ID: 1EVE). The three peptides stably accommodated within the active site gorge of AChE, as shown in Figure 4A–C. The interaction heatmap (Figure 4D) illustrated that hydrophobic interactions served as the primary driving force for peptide binding, accompanied by hydrogen bonding, electrostatic salt bridges, and aromatic π–π stacking. Residue contact analysis (Figure 4E) identified critical interaction residues, including ASP72, TRP84, TYR121, PHE331, and TYR334. Specifically, TRP84 and PHE331 participated in stable hydrophobic and aromatic stacking with the peptides, while ASP72, TYR121, and TYR334 formed polar contacts and hydrogen bonds, primarily within the peripheral anionic site (PAS) of the enzyme. Donepezil was used as a positive control. The binding energy score of E2020 docked into the AChE active site was −11.79 ± 1.93 kcal/mol. The specific conformation is shown in Figure S2.

### 2.5. Structural and Thermodynamic Stability via Molecular Dynamics (MD) Simulations

**Figure 5:**
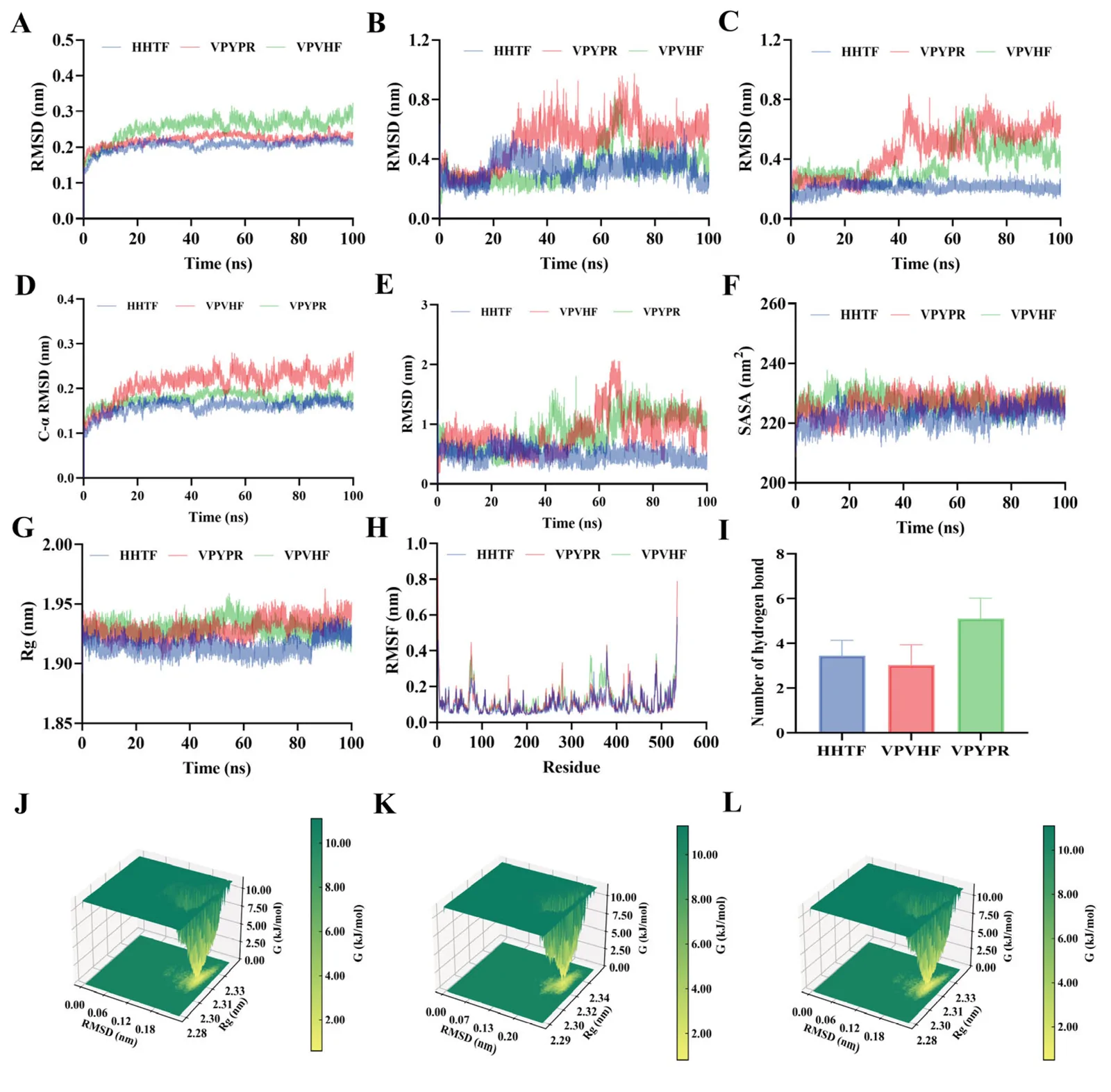
MD simulations of the three peptide–AChE complexes. (**A**) RMSD of the complexes. (**B**) RMSD in the CAS region. (**C**) RMSD in the PAS region. (**D**) C-alpha atom RMSD. (**E**) RMSD of the ligand relative to the protein. (**F**) SASA. (**G**) Rg. (**H**) RMSF. (**I**) Number of hydrogen bonds between peptides and AChE. (**J**) Gibbs free energy landscape of HHTF–AChE. (**K**) VPVHF–AChE. (**L**) VPYPR–AChE. Abbreviations: MD, molecular dynamics; RMSD, root-mean-square deviation; CAS, catalytic anionic site; PAS, peripheral anionic site; SASA, solvent-accessible surface area; Rg, radius of gyration; RMSF, root-mean-square fluctuation.

To evaluate complex stability under near-physiological conditions, 100 ns MD simulations were performed. The structural thermodynamic parameters equilibrated successfully (Figure S1). Backbone root-mean-square deviation (RMSD) values converged to stable plateaus, yielding average values of 0.22 ± 0.03 nm for HHTF–AChE, 0.24 ± 0.02 nm for VPVHF–AChE, and 0.27 ± 0.04 nm for VPYPR–AChE (Figure 5A). Regional RMSD analyses of the catalytic anionic site (CAS) and peripheral anionic site (PAS) revealed lower structural fluctuations in the PAS region compared to the CAS region across all three complexes (Figure 5B,C).

To more rigorously characterize the stability of the receptor and the dynamic behavior of the peptides in the binding pocket, the C-alpha RMSD of the protein and the ligand RMSD relative to the protein backbone were analyzed separately over the 100 ns trajectories (Figure 5D,E). As shown in Figure 5D, the C-alpha RMSD values of the protein in all three complexes rapidly converged within the initial 10 ns and remained stable below 0.28 nm throughout the simulation, confirming that the receptor maintains a stable tertiary structure without significant denaturation upon peptide binding. Notably, the ligand RMSD relative to the protein (Figure 5E) revealed distinct binding dynamics among the three peptides: HHTF exhibited the lowest and most stable RMSD, indicating that it is tightly anchored in the active site with minimal conformational deviation. In contrast, VPVHF and VPYPR showed higher relative RMSD values, reflecting larger conformational rearrangements or spatial flexibility within the binding groove. These results are consistent with the higher binding affinity observed for HHTF.

The solvent-accessible surface area (SASA) and radius of gyration (Rg) remained highly stable throughout the trajectories (Figure 5F,G). Average SASA values were 224.33 ± 4.01 nm^2^ (HHTF), 226.31 ± 5.42 nm^2^ (VPVHF), and 226.76 ± 6.60 nm^2^ (VPYPR), while average Rg values equilibrated at 2.52 ± 0.01 nm (HHTF) and 2.71 ± 0.02 nm (VPVHF and VPYPR). Root mean square fluctuation (RMSF) analysis (Figure 5H) confirmed that local residues experienced limited fluctuations (0.1–0.5 nm).

Furthermore, statistical analysis of three independent replica simulations (*n* = 3) demonstrated persistent intermolecular hydrogen-bond networks (Figure 5I). Among the three complexes, VPYPR formed the highest average number of hydrogen bonds (5.10 ± 0.92), higher than that of HHTF (3.45 ± 0.69) and VPVHF (3.03 ± 0.91). Gibbs free energy landscapes (Figure 5J–L) showed that all three complexes converged toward well-defined, low-energy basins.

### 2.6. Molecular Mechanics Poisson–Boltzmann Surface Area (MM-PBSA) Binding Free Energy Calculations

**Figure 6:**
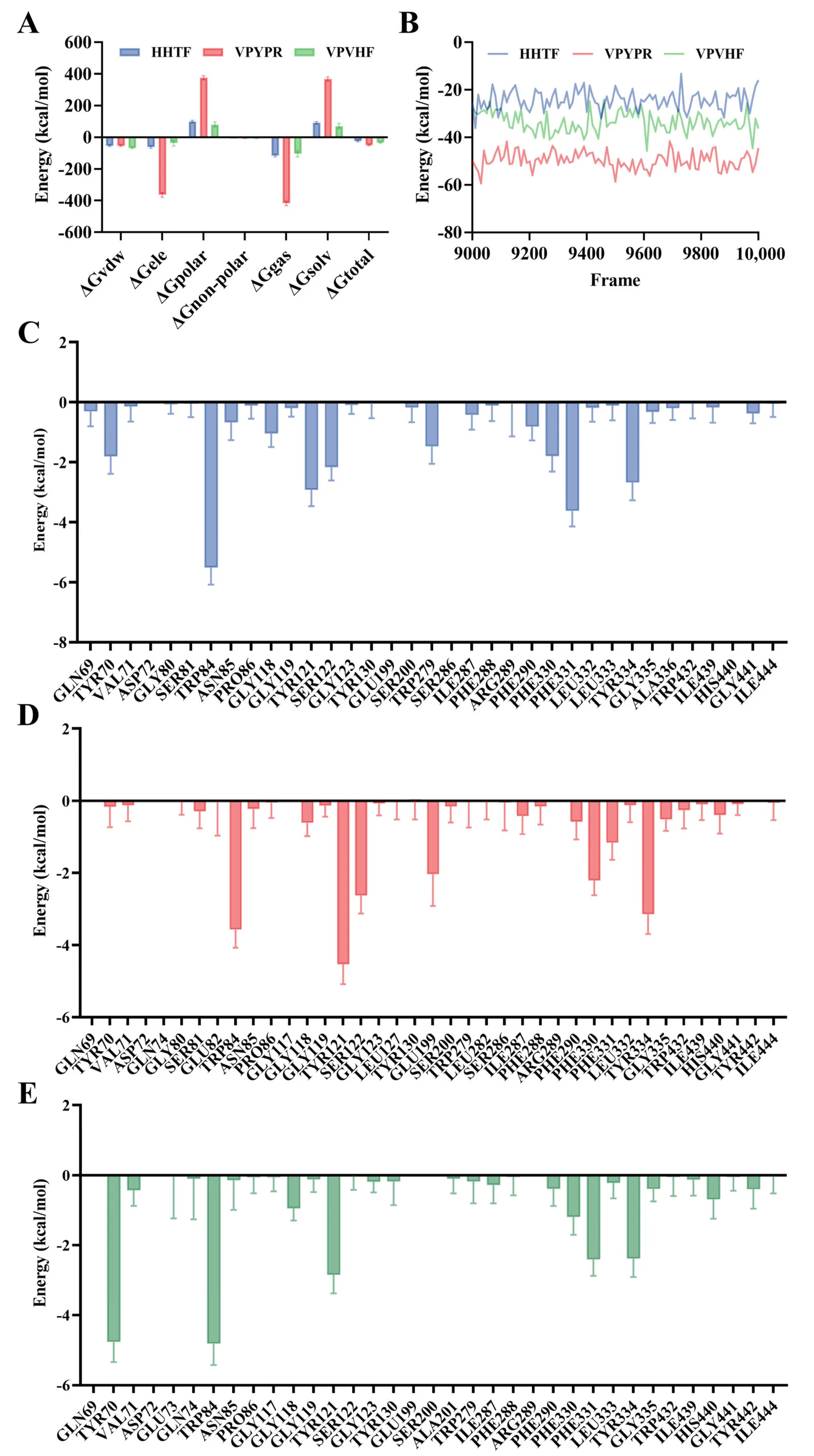
MM-PBSA analysis. (**A**) Total energy decomposition of peptide–AChE complexes. (**B**) Binding free energy over simulation time. (**C**) Residue energy contributions for HHTF–AChE. (**D**) VPYPR–AChE. (**E**) VPVHF–AChE. Abbreviations: MM-PBSA, molecular mechanics Poisson–Boltzmann surface area; AChE, acetylcholinesterase; Δ Gtotal, total binding free energy; Δ Gvdw, van der Waals energy; Δ Gele, electrostatic energy; Δ Gpolar, polar solvation energy; Δ Ggas, gas-phase energy; Δ Gsolv, solvation free energy.

Binding free energy calculations using the MM-PBSA method on equilibrated MD trajectories are shown in Figure 6A. All three complexes exhibited negative total binding free energies (Δ Gtotal). The VPYPR–AChE complex possessed the lowest binding free energy (−54.35 ± 4.69 kcal/mol), followed by VPVHF–AChE (−44.91 ± 5.20 kcal/mol) and HHTF–AChE (−35.37 ± 6.28 kcal/mol).

Energy decomposition revealed that van der Waals interactions (Δ Gvdw) were the primary favorable driving force across all complexes (Figure 6A). Electrostatic interactions (Δ Gele) also contributed favorably, exhibiting a pronounced impact in the VPYPR–AChE complex (Figure 6A). Polar solvation energy (Δ Gpolar) acted as the main unfavorable thermodynamic barrier, which was offset by favorable gas-phase energies (Δ Ggas) and non-polar solvation terms (Δ Gnon-polar). The temporal binding free energy profiles fluctuated within stable ranges (Figure 6B). Residue-wise energy decomposition (Figure 6C–E) identified TRP84, TYR121, PHE331, and TYR334 as key energetic contributors, demonstrating high consistency with the molecular docking analysis.

### 2.7. Precursor Protein Traceability

Traceability analysis within the *Crassostrea* genus identified distinct precursor origins for the seven selected peptides. Tripeptides HWF and KYW matched multiple precursors (248 and 234 protein hits, respectively). In contrast, pentapeptides VPVHF and GPKPW showed high sequence specificity, matching only 5 and 17 proteins, respectively. VPYPR was predominantly traced to structural α-tubulin proteins. GPKPW was linked to fructose–bisphosphate aldolase, whereas VPVHF and PVHF mapped to peroxisomal ATPase PEX1 and transport-associated proteins. HHTF was traced back to highly conserved actin and actin-like cytoskeletal proteins.

To further elucidate the enzymatic mechanism of simulated gastrointestinal proteolysis, the cleavage motif across the P4–P4’ region was mapped from all identified sequences (Figure S4). The motif revealed distinct enrichment of hydrophobic residues (Leu, Phe) and basic residues (Lys, Arg) adjacent to the cleavage junction, which aligns well with the cleavage profiles of pepsin and trypsin and explains the abundant release of bioactive oligopeptides containing terminal hydrophobic and basic amino acids.

## 3. Discussion

### 3.1. Nutritive, Structural, and Physicochemical Advantages of Oyster Digests

The simulated gastrointestinal digestion of *C. gigas* demonstrated high proteolytic efficiency, successfully converting intact marine proteins into low-molecular-weight peptide fractions. The static digestion protocol adopted from Brodkorb et al. [12] allowed for a standardized and reproducible evaluation of protein degradation products. The enrichment of short peptides (<2000 Da, representing 84.18% of the digests) is biologically advantageous. As highlighted by Xu et al. [13] and Karami et al. [14], low-molecular-weight oligopeptides (especially those within 180–1000 Da) exhibit superior solubility, enhanced intestinal absorption via peptide transporters (such as PepT1), and improved bioaccessibility to target organs compared to larger intact proteins or polypeptides. From a nutritional perspective, the high proportion of acidic residues (glutamic and aspartic acids, 28.5%) contributes both to the characteristic umami taste profile and to vital cellular metabolic processes [15,16,17].

Crucially, the digests contained substantial levels of basic (Arg, Lys, His) and hydrophobic amino acids. Basic amino acid side chains are often protonated under physiological pH, providing a positive charge that facilitates electrostatic attraction toward negatively charged biomolecules. Hydrophobic residues promote interactions with lipid cell membranes, facilitate entry into the hydrophobic binding pockets of enzymes, and enhance radical-scavenging capacities [18,19]. The abundance of hydrophobic and basic residues across pentapeptides and hexapeptides suggests that gastrointestinal digestion of *C. gigas* naturally yields structural motifs optimized for bioactivity and enzyme recognition.

### 3.2. Efficiency of the Integrated Computational–Experimental Bioprospecting Platform

Traditional bioactive peptide discovery relies heavily on activity-guided fractionation and trial-and-error experimental screening, which are inherently time-consuming, resource-intensive, and low-throughput [11,20]. In this study, we employed an integrated peptidomics and in silico screening pipeline that dramatically accelerated the bioprospecting process, filtering 18,292 identified sequences down to seven high-potential candidates. The utilization of multi-stage virtual screening tools (including abundance, pI, PeptideRanker, safety, and blood–brain barrier predictions) ensured that the synthesized peptides possessed promising drug-like qualities [21].

The prediction of BBB permeability is particularly vital for neuroprotective agents targeting the central nervous system [22]. Highly active molecules are of little therapeutic value in vivo if they cannot cross the blood–brain barrier to reach neuronal targets [23,24]. The favorable BBB scores obtained for VPYPR, VPVHF, and HHTF (Table 1) indicate that their short length and specific hydrophobic–basic balance are structurally compatible with brain delivery. Furthermore, the predicted non-toxicity was preliminarily supported by the absence of detectable cytotoxicity in PC12 cells at concentrations up to 200 μM. Nevertheless, BBB permeability, allergenicity, and systemic safety require further experimental validation.

### 3.3. Dual-Functional Neuroprotective Mechanisms: Balancing Oxidative Stress and Cholinergic Homeostasis

Oxidative stress and cholinergic dysfunction are two central, inextricably linked pathological contributors to neurodegenerative disorders like Alzheimer’s disease [2,3,4]. Excessive accumulation of reactive oxygen species (ROS) induces lipid peroxidation and compromises mitochondrial integrity, which in turn accelerates cholinergic degradation and neuronal dysfunction [4]. Conversely, impaired cholinergic transmission exacerbates oxidative vulnerability. While most previously reported marine peptides exhibit only single-target antioxidant properties, the oyster-derived peptides discovered in this study (HHTF, VPYPR, and VPVHF) demonstrated a dual-functional neuroprotective mechanism.

In the oxidative stress model, the peptides significantly rescued PC12 cells from H_2_O_2_-induced damage [25,26]. Mechanistically, this cytoprotection was achieved by reinforcing the endogenous enzymatic antioxidant system (significantly elevating SOD activity) and restoring the non-enzymatic cellular defense pool (increasing GSH levels) [27,28]. The concurrent reduction in MDA, a marker of lipid peroxidation, confirmed that the peptides preserved membrane integrity against ROS attack. In this study, acetylcholinesterase (AChE) was selected as the primary target due to its predominant catalytic role in synaptic acetylcholine clearance and its established clinical validation. While broader cholinesterase profiling—including Butyrylcholinesterase (BChE) and dual-acting targets—will provide deeper insights into selectivity and multi-target potential, evaluating AChE served as the primary proof-of-concept for the binding affinity and mechanism of these novel derivatives.

Simultaneously, the three peptides effectively normalized H_2_O_2_-induced abnormal AChE elevation [2]. Among them, VPYPR exhibited the most potent regulatory effect, restoring intracellular AChE activity to a level that was not significantly different from the normal control group. Donepezil restored intracellular AChE activity to the normal-control level, confirming the responsiveness of the cellular model to a clinically established AChE inhibitor. Among the tested peptides, VPYPR produced the closest cellular AChE response to that of donepezil, although direct enzyme-inhibition kinetics and IC_50_ values are required before their inhibitory potencies can be quantitatively compared. This outstanding performance can be attributed to its unique sequence structure: a pentapeptide containing a hydrophobic N-terminus (Val), rigid Proline residues, an aromatic Tyrosine, and a basic C-terminus (Arg). While Pro restricts backbone flexibility to reduce entropy loss upon binding [29], Arg and Tyr facilitate crucial polar, electrostatic, and aromatic interactions with the enzyme [30]. This dual antioxidant and cholinergic-regulatory action of oyster peptides represents a highly attractive multi-target therapeutic strategy for cognitive health maintenance [23].

### 3.4. Structural and Thermodynamic Basis of AChE Inhibition via PAS Association

Molecular docking and MD simulations provided deep atomistic insights into how these peptides interact with AChE. The active site gorge of AChE is characterized by a narrow, 20 Å-deep aromatic cavity that guides the acetylcholine substrate toward the catalytic triad at the bottom [31,32]. Our docking and MD results revealed that HHTF, VPYPR, and VPVHF do not bind exclusively to the catalytic triad; instead, they stably associate near the mouth of the gorge, interacting with residues of the peripheral anionic site (PAS), such as ASP72, TYR121, and TYR334.

Binding to the PAS is highly advantageous [32]. PAS-targeting ligands can sterically block substrate entry into the catalytic gorge and induce allosteric changes that suppress catalytic activity. Furthermore, the PAS of AChE is known to accelerate amyloid-beta (Aβ) aggregation. Because AChE PAS has been implicated in Aβ aggregation, whether these peptides affect PAS-associated Aβ aggregation warrants further investigation; this effect was not evaluated in the present study.

The 100 ns MD simulations confirmed the high stability of these peptide–AChE complexes, with low RMSD fluctuations (≤0.26 nm) and stable Rg values. The lower RMSD values observed in the PAS region compared to the CAS region further supported the thermodynamic preference for outer-gorge binding. VPYPR ranked first in both the MM-PBSA analysis and the cellular AChE assay, whereas the relative ranking of HHTF and VPVHF differed between the computational and cellular results.

Thermodynamic decomposition revealed that van der Waals forces and hydrophobic packing (particularly with TRP84 and PHE331) are the primary drivers of binding [33]. However, VPYPR’s significantly superior binding affinity is due to its highly favorable electrostatic contribution (−354.2 kcal/mol), driven by its terminal arginine residue forming salt bridges with ASP72 and surrounding polar residues. This combination of hydrophobic stabilization and electrostatic anchoring explains why VPYPR serves as an exceptionally effective AChE regulator [34,35].

Although the crystal structure of *Torpedo californica* AChE (PDB ID: 1EVE) was employed for docking and MD simulations owing to its benchmark status, multiple sequence alignment with rat (*Rattus norvegicus*, UniProt: P37136) and human (*Homo sapiens*, UniProt: P22303) AChE demonstrates that the active site gorge is strictly conserved. The structures of the three proteins are shown in Figure S3. Specifically, the key binding residues identified in our computational models—Asp72, Trp84, Tyr121, Phe331, and Tyr334 in *T. californica*—correspond directly to Asp74, Trp86, Tyr124, Phe338, and Tyr341 in mature rat and human AChE. This structural identity confirms that our in silico findings accurately reflect the inhibitory mechanisms observed in the rat PC12 cellular assay.

### 3.5. Biological Traceability, Physiological Significance, and Future Perspectives

Protein origin analysis verified that the identified candidates were not random artifacts but were rationally released from abundant structural and metabolic proteins of *C. gigas*. The release of HHTF from actin and VPYPR from alpha-tubulin is consistent with the rich abundance of cytoskeletal proteins in oyster muscle tissues and their susceptibility to gastric and pancreatic proteases [36,37]. Interestingly, PVHF and VPVHF shared identical precursor origins, indicating that PVHF is a truncated, intermediate digest of VPVHF, further demonstrating the progressive nature of gastrointestinal hydrolysis.

The high sequence specificity of VPVHF and GPKPW, which matched only a few precursor proteins, makes them potential biomarker candidates for tracking oyster protein digestion in vivo [38]. While our computational and cellular results are promising, future studies should focus on verifying the gastrointestinal stability of these peptides using ex vivo intestinal transport models and validating their neuroprotective efficacy in vivo using animal models of cognitive impairment. Nevertheless, the dual-functional neuroprotective profile and high biocompatibility of these Pacific oyster-derived peptides highlight their strong potential as functional food ingredients and marine-derived nutraceuticals for mitigating age-related neurodegenerative decline.

## 4. Materials and Methods

### 4.1. Materials and Reagents

Pacific oysters (*Crassostrea gigas*) with a shell length of 8–12 cm were collected from a commercial breeding farm in Rushan, Shandong Province, China. Pepsin (CAS: 9001-75-6) and trypsin (CAS: 9002-07-7) were purchased from Shanghai Yuanye Bio-Technology Co., Ltd. (Shanghai, China). All experiments were performed using HPLC-grade and mass spectrometry-grade reagents. Assay kits for MDA (Iterm number: ADS-F-YH002-96), SOD (Item number: ADS-F-KY011-96), GSH (Item number: ADS-W-G003), and AChE (Item number: ADS-W-ZM001) were obtained from Jiangsu Aidisheng Biotechnology Co., Ltd. (Taizhou, Jiangsu, China). PC12 cells and their specific culture medium (Cat. No. TCR-G617) were purchased from Suzhou Haixing Biotechnology Co., Ltd. (Suzhou, China).

### 4.2. Simulated Gastrointestinal Digestion

Oyster samples were shucked and cleaned to remove impurities. The oyster meat was homogenized in an ice-water bath, and the lyophilized oyster powder was further homogenized to obtain a uniform sample. The simulated digestion protocol developed by Brodkorb et al. was adopted [12]. This method evaluates the digestive efficiency by measuring digestion products (e.g., peptides and amino acids) and the release of micronutrients from the food matrix. Simulated gastric fluid (SGF) and simulated intestinal fluid (SIF) were prepared prior to digestion. The processed oyster sample was mixed with water (1:10, *w*/*v*) until fully blended. To each 20 mL of the mixture, 7.2 mL of SGF, 0.04 mL of CaCl_2_ (0.3 M), 1.92 mL of pepsin (2000 U·mL^−1^), 0.44 mL of water, and 0.4 mL of HCl (1 M) were added. The digestion mixture was then incubated at 37 °C for 2 h.

After gastric digestion, the sample was neutralized to pH 6.5. The neutralized sample was then mixed with 12.8 mL of SIF, 0.04 mL of CaCl_2_ (0.3 M), 5 mL of trypsin (100 U·mL^−1^), and sufficient water to reach a final volume of 40 mL. The mixture was incubated at 37 °C for 2 h with continuous shaking. The reaction was terminated by adding formic acid.

### 4.3. Molecular Weight Distribution of Oyster Simulated Digesta

The simulated digesta (10 mg) was dissolved in 2 mL of 100% dimethyl sulfoxide (DMSO) containing 0.5% lithium bromide, followed by continuous stirring in a boiling water bath for 12 h. The molecular weight distribution was determined using a Dawn Heleos II high-performance size-exclusion chromatography system (Wyatt Technology, Santa Barbara, CA, USA), following the method of Zhao et al. with minor modifications [36].

### 4.4. Total Amino Acid Composition Analysis

The total amino acid composition of the simulated digests was determined following standard acid hydrolysis coupled with High-Performance Liquid Chromatography (HPLC). Briefly, the lyophilized sample was hydrolyzed with 6 M HCl at 110 °C for 24 h under vacuum. After cooling, the hydrolysate was filtered, diluted, and subjected to HPLC analysis for amino acid quantification. Data are expressed as the relative percentage of total amino acids.

### 4.5. Peptidomic Identification of Oyster Simulated Digesta

To account for biological variation, oysters were collected in three distinct biological batches (*n* = 3). Tissues from each batch were processed separately for protein extraction, enzymatic digestion, and subsequent LC-MS/MS analysis. The digested oyster sample was dissolved in 0.1% trifluoroacetic acid (TFA) aqueous solution. Desalting was performed using a C18 column (Waters Technologies Shanghai Ltd., Shanghai, China) as follows: the column was rinsed with acetonitrile and preconditioned with 0.1% TFA; then the peptide sample was loaded, and salts and impurities were removed with 0.1% TFA; finally, peptides were eluted with an aqueous solution containing 50% acetonitrile and 0.1% TFA. After overnight freeze-drying, the peptides were redissolved in 20 μL of 0.1% formic acid for subsequent LC-MS analysis.

The desalted samples were subjected to mass spectrometry. All MS parameters were set according to the method of Lan et al. [38]. Peptides were first trapped on a trap column for 3 min and then separated on a nano-analytical column using gradient elution. MS data analysis and protein database searching were performed using Peak Studio 8.5 (Bioinformatics Solutions, Waterloo, ON, Canada). The resulting identifications were filtered at a false discovery rate (FDR) of ≤1.0% at the peptide level, and redundant entries were collapsed to yield distinct peptide primary sequences for downstream virtual screening. The full peptide- and protein-level identification files across all biological replicates are available in the Supplementary Materials.

### 4.6. Virtual Screening of Bioactive Peptides

The peptides identified via peptidomics were sequentially screened using online tools: PeptideRanker (https://peptide.ucd.ie/peptideranker, accessed on 24 April 2026), DeepB^3^P (https://cbcb.cdutcm.edu.cn/deepb3p/, accessed on 24 April 2026), Expasy-protParam (https://web.expasy.org/compute_pi/, accessed on 24 April 2026), ToxinPred (https://webs.iiitd.edu.in/raghava/toxinpred/, accessed on 24 April 2026), AllerMatch (https://allermatch.org/allermatchsearch/form, accessed on 24 April 2026), and Innovagen (https://www.innovagen.com/tools/, accessed on 24 April 2026) [39,40,41,42,43,44]. These tools were used to predict peptide bioactivity, blood–brain barrier (BBB) permeability, isoelectric point (pI), toxicity, and allergenicity.

### 4.7. Molecular Docking

The three-dimensional conformations of the peptides were generated based on their sequences using RDKit (version 2022.9.5) implemented in Python 3.11.4 [25]. The MMFF94 and ETKDG force fields within RDKit were used to construct the peptide conformations. After energy minimization, the MOL files containing peptide structures were converted to PDB format using OpenBabel (3.1.1). The crystal structure of AChE (PDB ID: 1EVE) was obtained from the RCSB Protein Data Bank [21]. This model was selected due to its high resolution and the evolutionary conservation (>85% identity) of the active gorge between *T. californica* and mammalian AChE. Using PyMOL (version 3.0.0 Schrödinger, LLC, New York, NY, USA), water molecules and co-crystallized ligands were removed, hydrogen atoms were added, and the protein was set as a rigid receptor model. To map key binding contacts onto rodent AChE, sequence alignment was performed across *T. californica* (1EVE), rat (*R. norvegicus*, P37136), and human (*H. sapiens*, P22303) AChE using NCBI BLASTp web server (https://blast.ncbi.nlm.nih.gov/Blast.cgi, accessed on 14 August 2026) and the EMBL-EBI Clustal Omega web server (version 1.2.4, https://www.ebi.ac.uk/jdispatcher/msa/clustalo, accessed on 14 August 2026). Subsequently, AutoDockTools-1.5.6 was used to merge non-polar hydrogen atoms, assign Gasteiger charges, and remove lone pair electrons from both the ligand and receptor. Molecular docking was performed using AutoDock Vina (1.2.0). The binding site of AChE was defined by selecting residues surrounding the native ligand in the E2020 structure, with the grid box centered at coordinates x = 2.78, y = 64.38, z = 67.97, and a radius of 9.15 Å.

### 4.8. PC12 Cell Culture

PC12 cells were cultured in RPMI 1640 medium supplemented with 10% (*v*/*v*) fetal bovine serum, 100 U/mL penicillin, and 100 μg/mL streptomycin at 37 °C in a 5% CO_2_ incubator. To ensure statistical rigor and reproducibility, all quantitative cell-based assays (including cell viability, cytotoxicity, and protein expression assays) were conducted with at least three independent biological replicates (N ≥ 3), defined as separate experiments performed on distinct days using different cell passage batches. Within each biological replicate, all treatment conditions and controls were tested in triplicate or quadruplicate (*n* ≥ 3 technical replicates per plate). The cytotoxicity assay was performed following the method of Morshedi et al. with minor modifications [29]. When cell confluence reached 90%, a single-cell suspension was prepared, and cells were counted. Cells were seeded in 96-well plates at a density of 2 × 10^4^ cells per well and cultured for 24 h. The medium was then replaced with 100 μL of RPMI 1640 basal medium (control) or 100 μL of basal medium containing different peptide concentrations (experimental groups). A blank group (no cells) was also included.

After 24 h of incubation, 10 μL of Cell Counting Kit-8 (CCK-8) solution was added to each well, and the cells were incubated for an additional 1 h. Absorbance was measured at 450 nm, and cell viability was calculated as follows:

Cell viability (%) = OD_Experimental group_ − OD_Blank group_/OD_Control group_ − OD_Blank group_ × 100% (1)

To establish an oxidative stress model, PC12 cells were seeded in 96-well plates. A blank group (basal medium only), a control group (cells in basal medium), and model groups (treated with H_2_O_2_ for 1 h) were designed [30]. Cells were exposed to various concentrations of H_2_O_2_ (100, 200, 300, 400, 500, 600, and 700 μM). After treatment, 10 μL of CCK-8 solution was added, and the cells were incubated for 1 h. Absorbance was measured at 450 nm, and cell viability was calculated using Formula (1). The H_2_O_2_ concentration that reduced cell viability to approximately 50% was selected for subsequent experiments.

After determining the optimal modeling conditions, PC12 cells were seeded in 96-well plates and cultured for 24 h. A control group (basal medium) and experimental groups were set up. The experimental groups included a model group receiving H_2_O_2_ alone, a positive-control group pretreated with 1 μM donepezil, and peptide groups pretreated with 50, 100, or 200 μM peptide. After 24 h of peptide treatment, the cells were exposed to the predetermined concentration of H_2_O_2_ for 1 h. Then, 10 μL of CCK-8 solution was added, and the cells were incubated for another 1 h. Absorbance was measured at 450 nm, and cell viability was calculated using Formula (1) to evaluate the protective activity of the peptides.

Additionally, PC12 cells at >90% confluence were seeded in 6-well plates at a density of 2 × 10^5^ cells per well. After 24 h, cells were treated according to the grouping described above, followed by H_2_O_2_ exposure for 1 h. The cells were then harvested by means of ice-bath sonication, and the supernatants were collected for the determination of GSH, SOD, AChE activity, and MDA content. All assays were performed according to the manufacturer’s instructions of the respective kits.

### 4.9. MD Simulation

The optimal conformations of protein–ligand complexes determined by means of molecular docking were used for subsequent MD simulations [44]. All MD simulations were performed using GROMACS (version 2020.06) with the CHARMM36 force field and the TIP3P water model [45,46]. Na^+^ and Cl^−^ ions were added to neutralize the system. Energy minimization was carried out using the steepest descent algorithm to remove unfavorable atomic interactions. Temperature equilibration (using the V-rescale method) and pressure equilibration (using the Parrinello–Rahman method) were then performed for 100 ps to stabilize the system at 300 K and 1 bar. The MD simulation of the protein–ligand complex system was run for 100,000 ps, with coordinates saved every 10 ps. Final data were extracted using built-in commands in GROMACS. To ensure statistical reliability and conformational convergence, all MD simulations were carried out in triplicate (3 × 100 ns).

### 4.10. MM-PBSA Binding Free Energy Calculation

Binding free energies were calculated using the MM-PBSA method on the equilibrated trajectories, extracted separately from the system after three parallel separations. The total binding free energy (ΔG bind) was decomposed into van der Waals, electrostatic, polar solvation, and non-polar solvation contributions. Residue-wise energy decomposition was performed to identify hotspot residues contributing most favorably to peptide–AChE binding.

### 4.11. Protein Origin Analysis

To verify the biological origin of selected peptides, protein traceability analysis was conducted using the UniProt database within the *Crassostrea* genus (https://www.uniprot.org/uniprotkb accessed on 14 August 2026). Precursor proteins for each peptide were identified, and the number of matched proteins was recorded as an indicator of sequence specificity. To characterize the enzymatic cleavage specificity of simulated gastrointestinal proteolysis, the cleavage motifs spanning the P4–P4’ region were mapped from all identified distinct peptide sequences according to the established peptidomic protocol [47,48]. Briefly, identified peptide sequences obtained from LC-MS/MS were merged with their corresponding full-length precursor protein sequences from the *Crassostrea gigas* UniProt reference proteome based on accession IDs. For each distinct cleavage junction, an octapeptide sequence window comprising four amino acid residues upstream of the scissile bond (designated conventionally as P4, P3, P2, and P1) and four residues downstream (designated as P1’, P2’, P3’, and P4’) was extracted. The position probability matrix (PPM) representing the frequency distribution of amino acids across the eight positions was determined, and the residue conservation and enrichment profiles were visualized as a stacked Sequence Logo using WebLogo 3.0/logomaker with information content reported in bits.

### 4.12. Data Analysis

All cellular experiments were independently repeated three times, with three technical replicates per treatment in each experiment. Technical replicates were averaged within each independent experiment, and the independent experiment was treated as the statistical unit (*n* = 3). Data are expressed as mean ± SD. Normality and homogeneity of variance were assessed using the Shapiro–Wilk and Levene tests, respectively. Multiple-group comparisons were performed using one-way ANOVA followed by Dunnett’s test for comparisons with the model group. Statistical significance was set at *p* < 0.05. Data visualization was carried out using GraphPad Prism 9.5.1 (GraphPad Software, San Diego, CA, USA) and Adobe Illustrator 2020 (Adobe Inc., San Jose, CA, USA).

## 5. Conclusions

In this study, we developed an integrated computational–experimental pipeline to identify AChE-targeting neuroprotective peptides from simulated gastrointestinal digests of Pacific oyster. From 18,292 peptidomic sequences, a multi-step in silico pipeline incorporating abundance filtering, basic residue selection, PeptideRanker scoring, BBB permeability prediction, and safety evaluation reduced the candidate pool to 65 peptides, with molecular docking against AChE further identifying seven candidates exhibiting strong binding affinities (−10.6 to −11.1 kcal/mol). Among these, VPYPR, VPVHF, and HHTF showed the most favorable interaction patterns. Over 100 ns MD simulations, all three complexes maintained stable binding (RMSD 0.20–0.26 nm) with persistent hydrogen bonding (>4 bonds on average), and lower fluctuations in the peripheral anionic site (PAS) region compared with the catalytic anionic site suggested preferential binding near the gorge entrance, involving residues TRP84, TYR121, PHE331, and TYR334. MM-PBSA binding free energy calculations quantitatively ranked the candidates, with VPYPR–AChE exhibiting the most favorable value (−49.74 ± 3.58 kcal/mol), followed by VPVHF–AChE (−33.65 ± 4.29 kcal/mol) and HHTF–AChE (−24.33 ± 3.95 kcal/mol); van der Waals interactions dominated the binding, with electrostatic contributions being particularly significant for VPYPR due to its terminal Arg residue. Experimental validation in H_2_O_2_-induced PC12 cells confirmed these computational predictions, as VPYPR, VPVHF, and HHTF dose-dependently restored GSH and SOD levels, reduced MDA accumulation, and suppressed abnormal AChE activity. VPYPR ranked first in both the cellular AChE assay and the MM-PBSA analysis, whereas the relative rankings of HHTF and VPVHF differed between the experimental and computational results. In summary, this work demonstrates that Pacific oyster is a promising dietary source of neuroprotective peptides and provides a computationally driven, experimentally validated pipeline for discovering marine-derived functional ingredients targeting cholinergic dysfunction and oxidative stress.

## Figures and Tables

**Table 1:** Sequences and predicted physicochemical and functional properties of the seven candidate peptides.

Sequence	pI	PeptideRanker Score	BBB Prediction Score	Toxin Prediction	Allergenicity Prediction	Docking Score (kcal/mol)
VPYPR	10.85	0.641232	1	Non-Toxin	Non-allergenic	−11.1
VPVHF	7.85	0.518013	1	Non-Toxin	Non-allergenic	−10.9
HHTF	8.00	0.50668	0.9999	Non-Toxin	Non-allergenic	−10.7
PVHF	7.85	0.718289	0.9997	Non-Toxin	Non-allergenic	−10.7
GPKPW	9.85	0.932373	0.7848	Non-Toxin	Non-allergenic	−10.6
HWF	7.85	0.990515	0.9984	Non-Toxin	Non-allergenic	−10.6
KYW	9.85	0.814066	0.9666	Non-Toxin	Non-allergenic	−10.6

Abbreviations: pI, isoelectric point; BBB, blood–brain barrier.

## Data Availability

The authors declare that the supporting data of this study are available within the article. The raw LC-MS/MS proteomics data have been deposited to the ProteomeXchange Consortium via the PRIDE repository (http://www.ebi.ac.uk/pride/archive/ accessed on 14 August 2026). Data are available via ProteomeXchange with identifier PXD082789.

## Supplementary Materials

The following supporting information can be downloaded at: https://www.mdpi.com/article/10.3390/md24090298/s1, Figure S1: Equilibrium parameters of the peptide–AChE complex at 100 ns ((A) Density, (B) Potential, (C) Pressure, (D) Temperature); Figure S2: Molecular docking of donepezil to AChE; Figure S3: 3D structures of the three proteins ((A) 1EVE, (B) P22303, (C) P37136); Figure S4: Proteolytic cleavage motif spanning the P4–P4’ region of peptides generated from simulated gastrointestinal digestion of Pacific oyster proteins. File S1 (MS&HPLC): Mass spectrometry (MS) and HPLC characterization of synthesized oyster AChE-targeting peptides and peptidomic mass spectra of the three candidate peptides identified after simulated gastrointestinal digestion.
