# Supplementary material for: Identification of Novel AChE-Targeting Neuroprotective Peptides from Pacific Oyster (Crassostrea gigas): An Integrated Pipeline of Peptidomics, Molecular Dynamics, and Cellular Validation
Source: Mar Drugs. 2026 Aug 25;24(9):298. doi: 10.3390/md24090298 (PMC13608502; doi:10.3390/md24090298)
Supplement: Supplementary file 1 [file marinedrugs-24-00298-s001.zip › oyster AChE peptide-Supplemental.pdf]

Identification of Novel AChE-Targeting Neuroprotective Peptides from Pacific Oyster (*Crassostrea gigas*): An Integrated Pipeline of Peptidomics, Molecular Dynamics, and Cellular Validation

Shi-Kun Suo [1], Kuo Dang [1], Ying-Ying Zhang [2, 3], Yao-Yao Zhang [2], Yu-Xin Luo [4],Jun-Wei Yan [2, 3], Dao-Dong Pan [1], Yan-Li Wang [1], Long Li [2, 3], Chao-Ying Zhang[5], Xin-Chang Gao [2, 5] *and Ya-Li Dang [1,*]

Author Affiliation (s)

[1] College of Food Science and Engineering, Ningbo University, Ningbo 315211, China; 2301390129@nbu.edu.cn (S.-K.S.); 2211390002@nbu.edu.cn (K.D.); daodongpan@163.com (D.-D.P.); wangyanli@nbu.edu.cn (Y.-L.W.)

[2] Institute of Drug Discovery Technology, Ningbo University, Ningbo 315211, China; 2411140327@nbu.edu.cn (Y.-Y.Z.); 2511130092@nbu.edu.cn (Y.-Y.Z.); 2411140323@nbu.edu.cn (J.-W.Y.);
lilong@nbu.edu.cn (L.L.)

[3] School of Pharmacy, Health Science Center, Ningbo University, Ningbo 315211, China

[4] Engineering Research Center of Storage and Processing of Xinjiang Characteristic Fruits and Vegetables, Ministry of Education, School of Food Science and Technology, Shihezi University, Shihezi 832000, China; luoyuxin@stu.shzu.edu.cn

[5] Department of Quality and Safety, Chinese Academy of Fishery Sciences, Beijing 100141, China; cyzhang0317@cafs.ac.cn

[6] Qian Xuesen Collaborative Research Center of Astrochemistry and Space Life Sciences, Ningbo University, Ningbo 315211, China

---

***Corresponding author:**

Xin-Chang Gao: gaoxinchang@nbu.edu,cn
Ya-Li Dang: dangyali@nbu.edu.cn

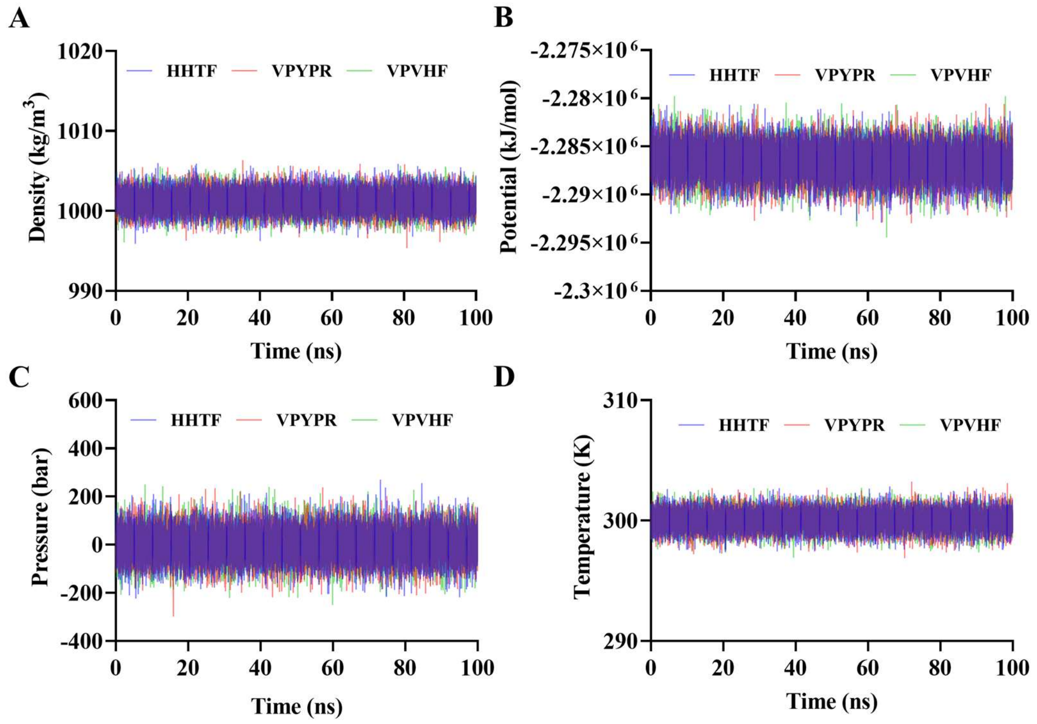

Figure S1. Equilibrium parameters of the peptide-AChE complex at 100 ns. (A) Density (B) Potential (C) Pressure (D) Temperature

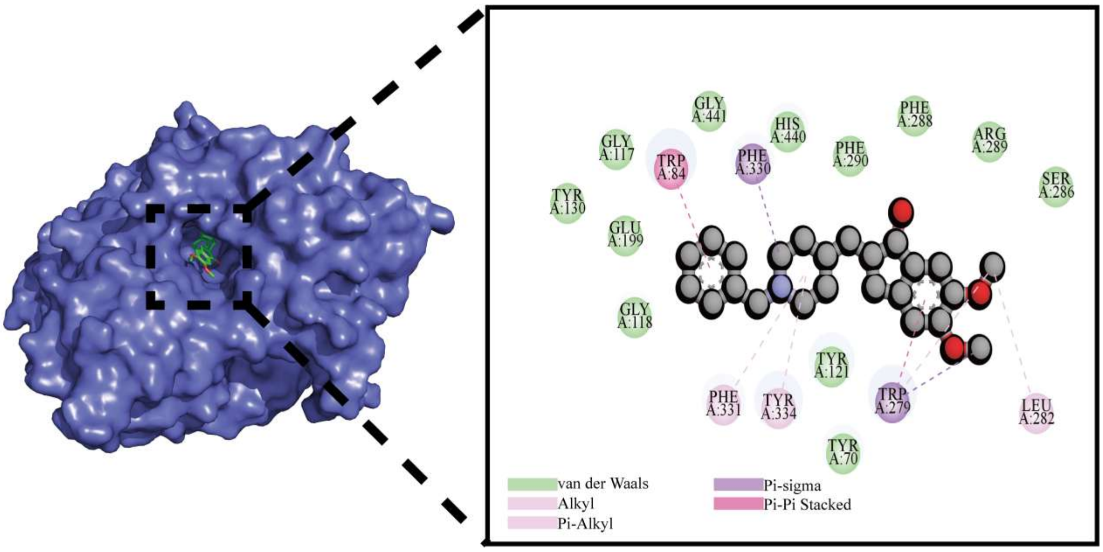

Figure S2. Molecular docking of donepezil to AChE.

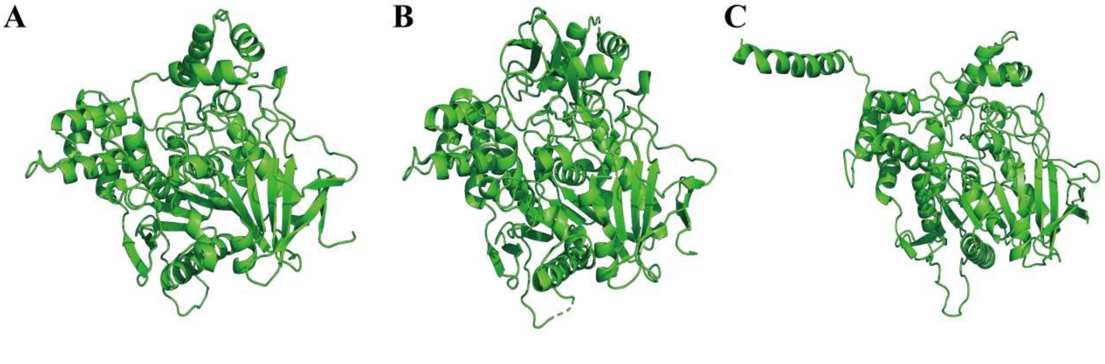

Figure S3. 3D structures of the three proteins. (A) 1EVE; (B) P22303; (C) P37136

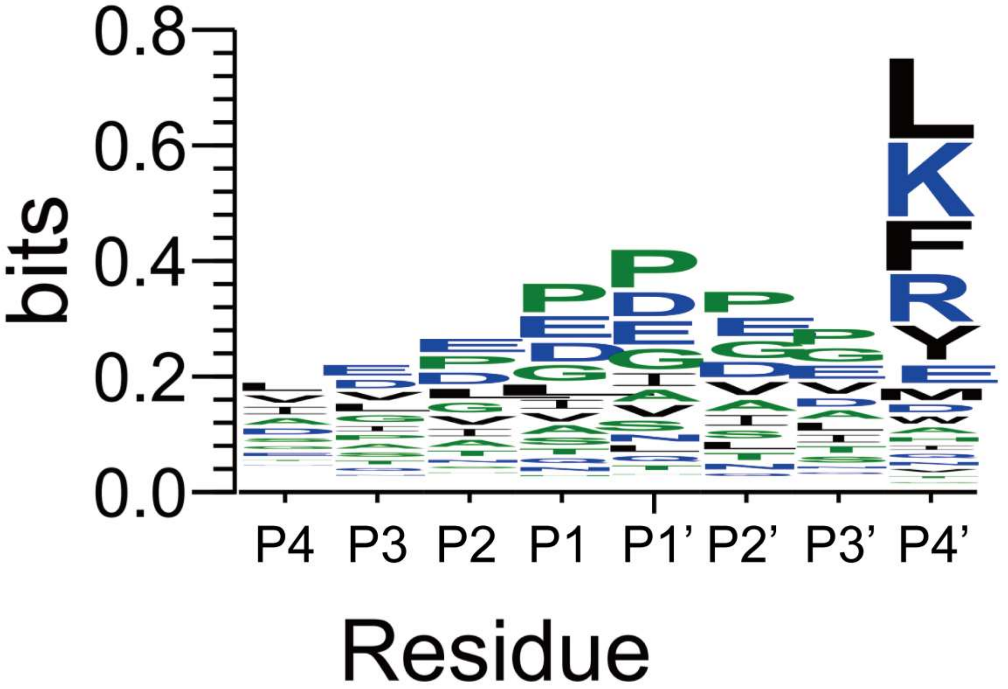

Figure S4. Proteolytic cleavage motif spanning the P4 – P4' region of peptides generated from simulated gastrointestinal digestion of Pacific oyster proteins. The height of each amino acid letter indicates its relative bit score (information content/frequency) at that specific position around the cleavage site.
