# Supplementary material for: Identification of Novel AChE-Targeting Neuroprotective Peptides from Pacific Oyster (Crassostrea gigas): An Integrated Pipeline of Peptidomics, Molecular Dynamics, and Cellular Validation
Source: Mar Drugs. 2026 Aug 25;24(9):298. doi: 10.3390/md24090298 (PMC13608502; doi:10.3390/md24090298)
Supplement: Supplementary file 1 [file marinedrugs-24-00298-s001.zip › 7-AChE peptides MS-HPLC.pdf]

HPLC REPORT

Sample: GPKPW

Analyzed Date: 2025-12-03

Analyst: Apeptide

Column: Boston Green 0DS-AQ, 4.6*250mm, 5μm

Solvent A A: 0.1% Trifluoroacetic Acid in 100% Acetonitrile

Solvent B B: 0.1% Trifluoroacetic Acid in 100% Water

Gradient :

	A	B
0.0min	15%	85%
25.0min	40%	60%
25.01min	100%	0%
	Stop	

Volume: 10μl

Wavelength: 220nm

Flow rate: 1.0ml/min

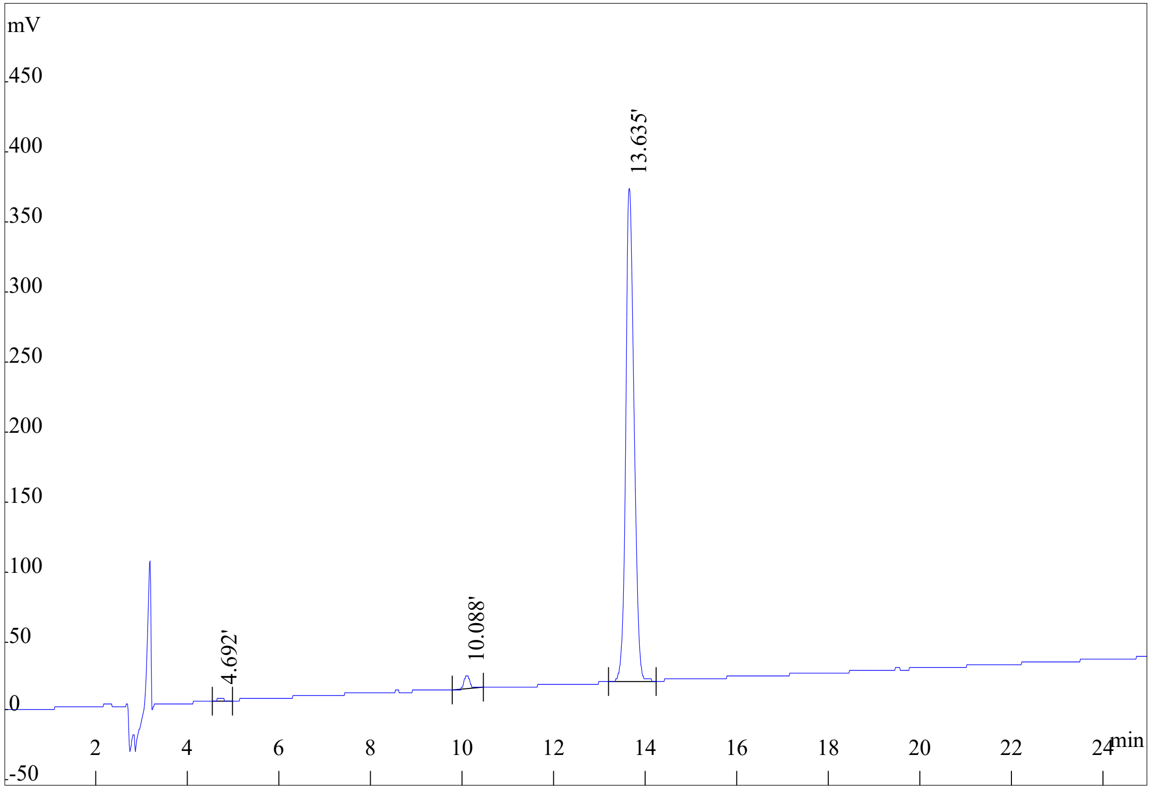

Rank	Time	Conc.	Area	Height
1	4.692	0.4031	17126	2189
2	10.088	1.887	80182	10217
3	13.635	97.71	4150902	351250
Total		100.0000		

MASS SPECTROMETRY REPORT

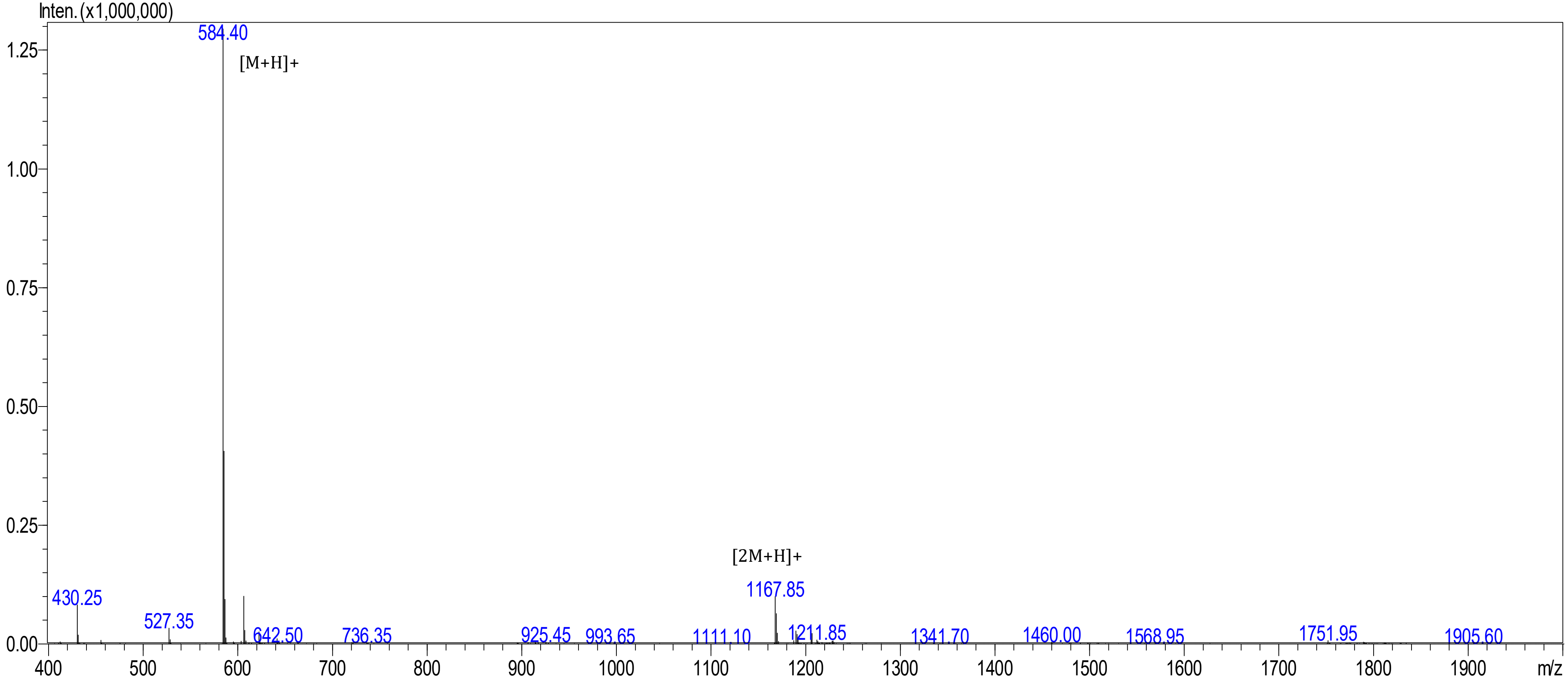

Sample Description		Instrument	SHIMADZU LCMS-2020		
Analyzed date:	2025-12-03	Probe:	ESI	Probe Bias:	＋4.5kv
Analyst:	Apeptide	Nebulizer Gas Flow:	1.5L/min	Detector:	1.5kv
Sample:	GPKPW	CDL:	-20.0v	T. Flow:	0.2ml/min
M.W.:	583.68	CDL Temp.:	250 ℃	B. Conc.:	50%H2O/50%ACN
Lot. No.:	Apep1308535	Block Temp.:	200 ℃		

HPLC REPORT

Sample:	HHTF	Analyzed date:	2025-12-01
Analyst:	Apeptide		
Column:	Symmetrix ODS-R, 4.6*250mm, 5µm		
Solvent A	A: 0.1% Trifluoroacetic Acid in 100% Acetonitrile		
Solvent B	B: 0.1% Trifluoroacetic Acid in 100% Water		

	A	B
0.0min	13%	87%
25.0min	38%	62%
25.1min	100%	0%
30.0min	Stop	

Volume:	20µl
Wavelength:	220nm
Flow rate:	1.0ml/min

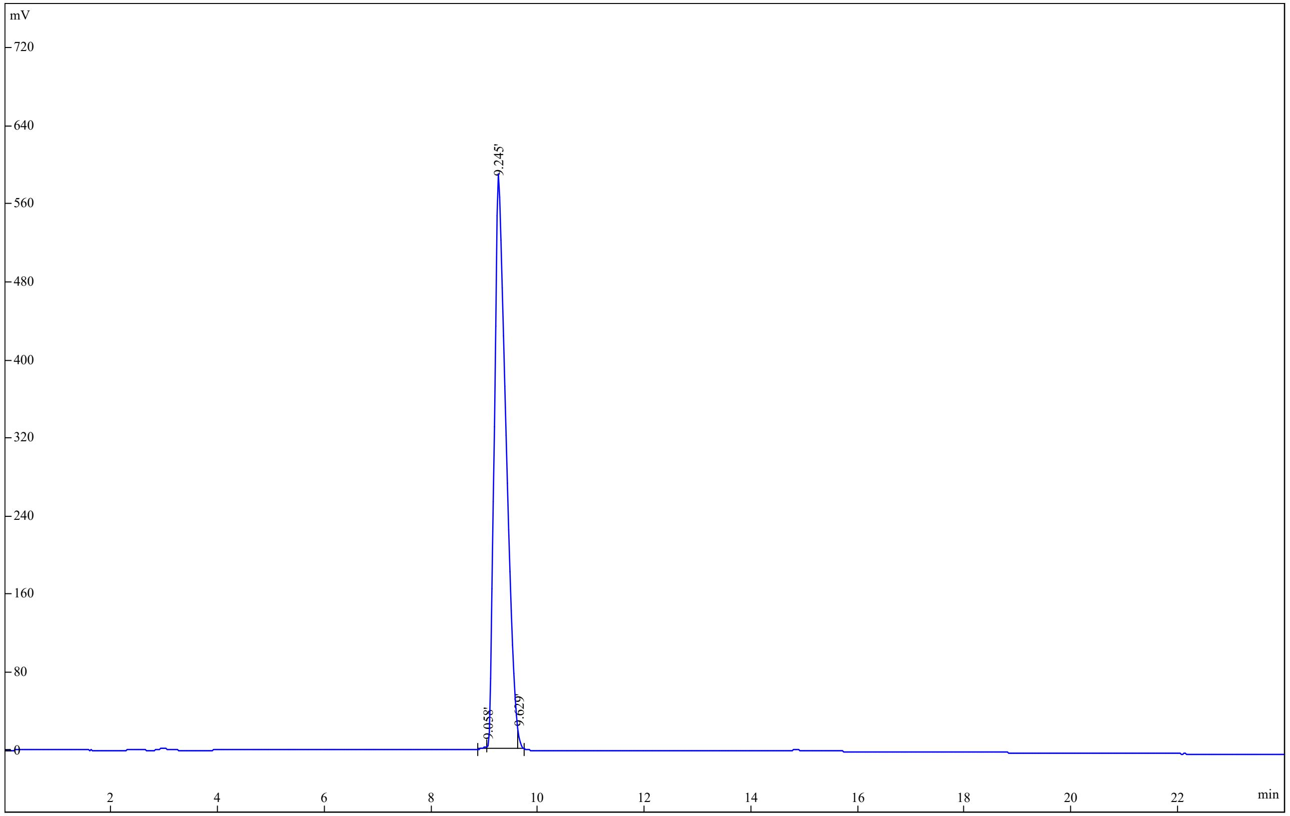

Rank	Time	Conc.	Area	Height
1	9.058	0.1738	15713	3965
2	9.245	99.52	8996402	591501
3	9.629	0.3136	28347	13322
Total		100	9040462	608788

MASS SPECTROMETRY REPORT

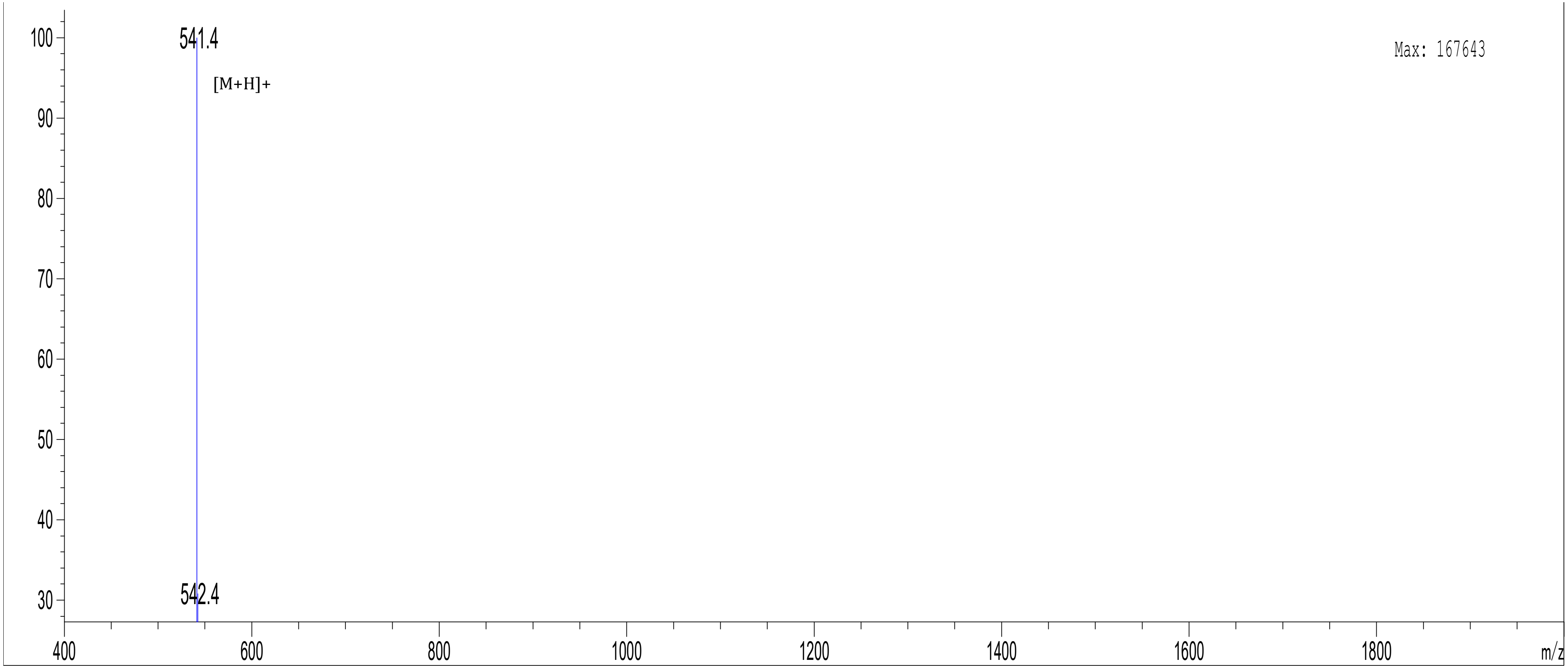

Sample Description		Instrument	Agilent-6125B		
Analyzed date:	2025-12-01	Probe:	ESI	Probe Bias:	＋4.5kv
Analyst:	Apeptide	Nebulizer Gas Flow:	1.5L/min	Detector:	1.5kv
Sample:	HHTF	CDL:	-20.0v	T. Flow:	0.2ml/min
M.W.:	540.57	CDL Temp.:	250 ℃	B. Conc.:	50%H2O/50%ACN
Lot. No.:	Apep1308533	Block Temp.:	200 ℃		

HPLC REPORT

Sample:	HWF	Analyzed Date:	2025-12-03
Analyst:	Apeptide		
Column:	Boston Green 0DS-AQ, 4.6*250mm, 5μm		
Solvent A	A: 0.1% Trifluoroacetic Acid in 100% Acetonitrile		
Solvent B	B: 0.1% Trifluoroacetic Acid in 100% Water		

Gradient :

	A	B
0.0min	20%	80%
25.0min	45%	55%
25.01min	100%	0%
	Stop	

Volume: 10μl

Wavelength: 220nm

Flow rate: 1.0ml/min

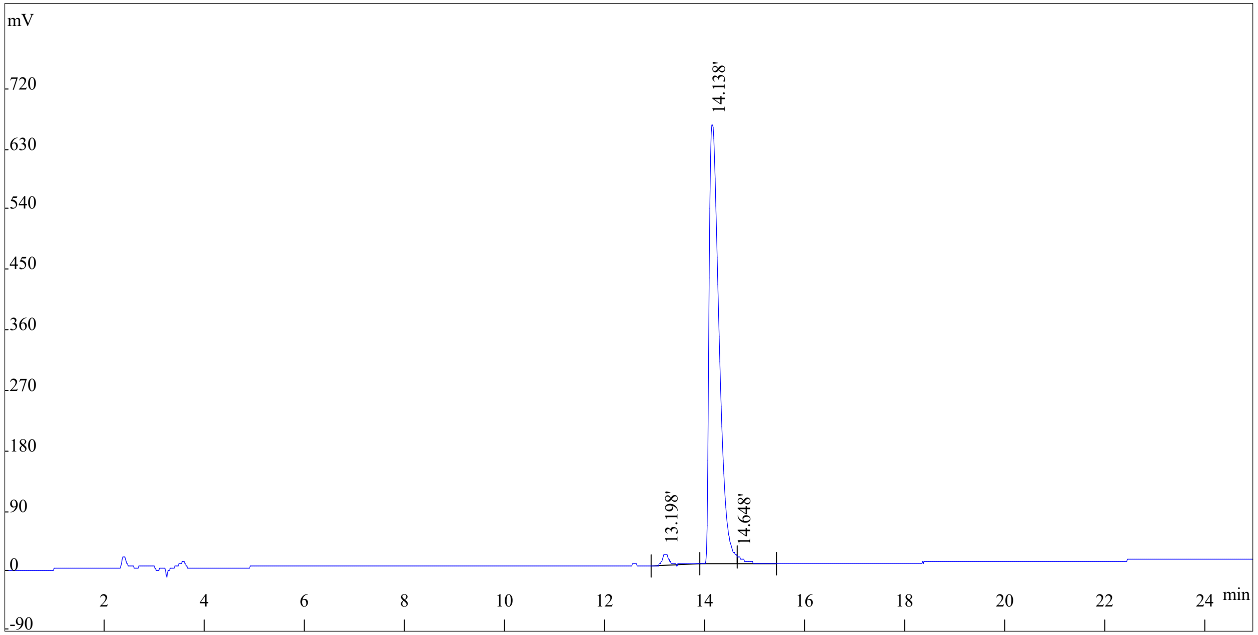

Rank	Time	Conc.	Area	Height
1	13.198	1.521	135200	16595
2	14.138	97.24	8641617	655720
3	14.648	1.236	109815	10788
Total		100.0000		

MASS SPECTROMETRY REPORT

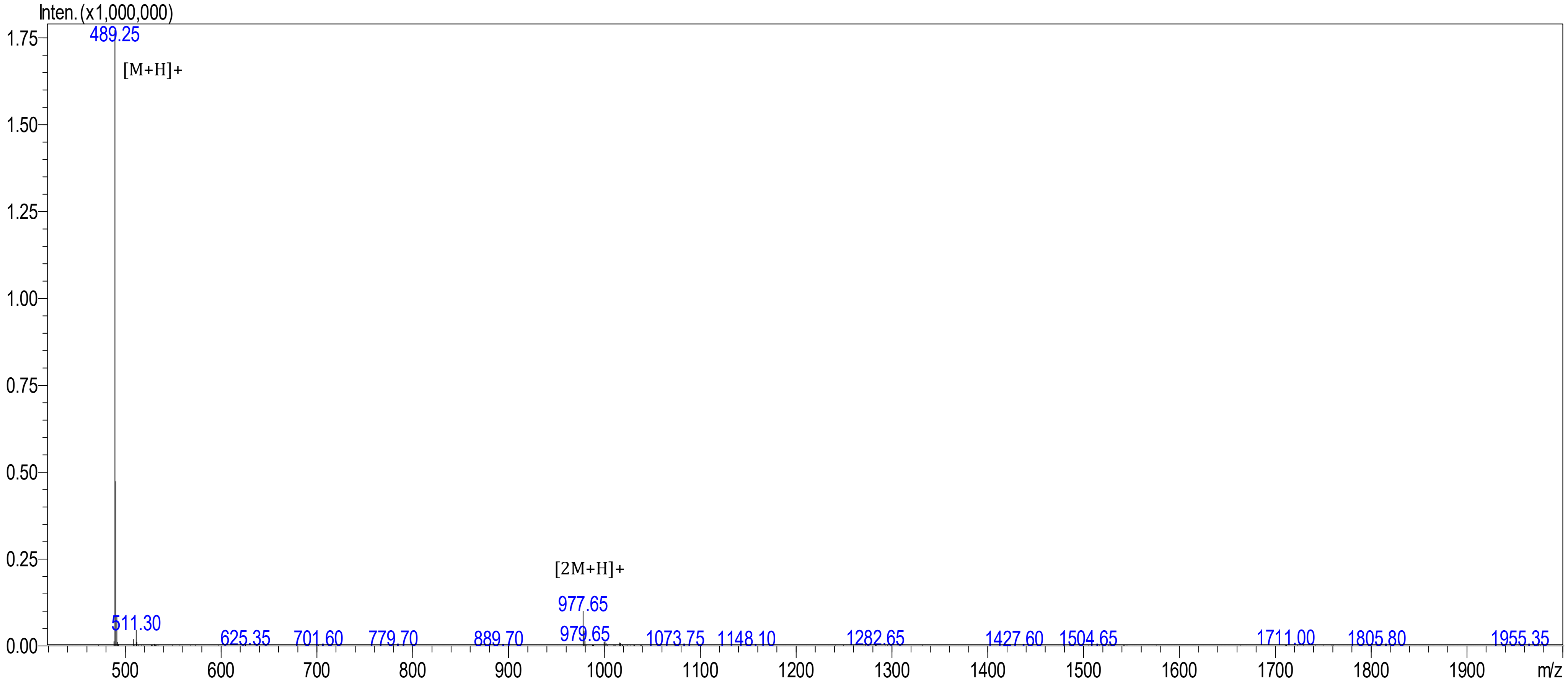

Sample Description		Instrument	SHIMADZU LCMS-2020		
Analyzed date:	2025-12-03	Probe:	ESI	Probe Bias:	＋4.5kv
Analyst:	Apeptide	Nebulizer Gas Flow:	1.5L/min	Detector:	1.5kv
Sample:	HWF	CDL:	-20.0v	T. Flow:	0.2ml/min
M.W.:	488.54	CDL Temp.:	250 °C	B. Conc.:	50%H2O/50%ACN
Lot. No.:	Apep250604	Block Temp.:	200 °C		

MASS SPECTROMETRY REPORT

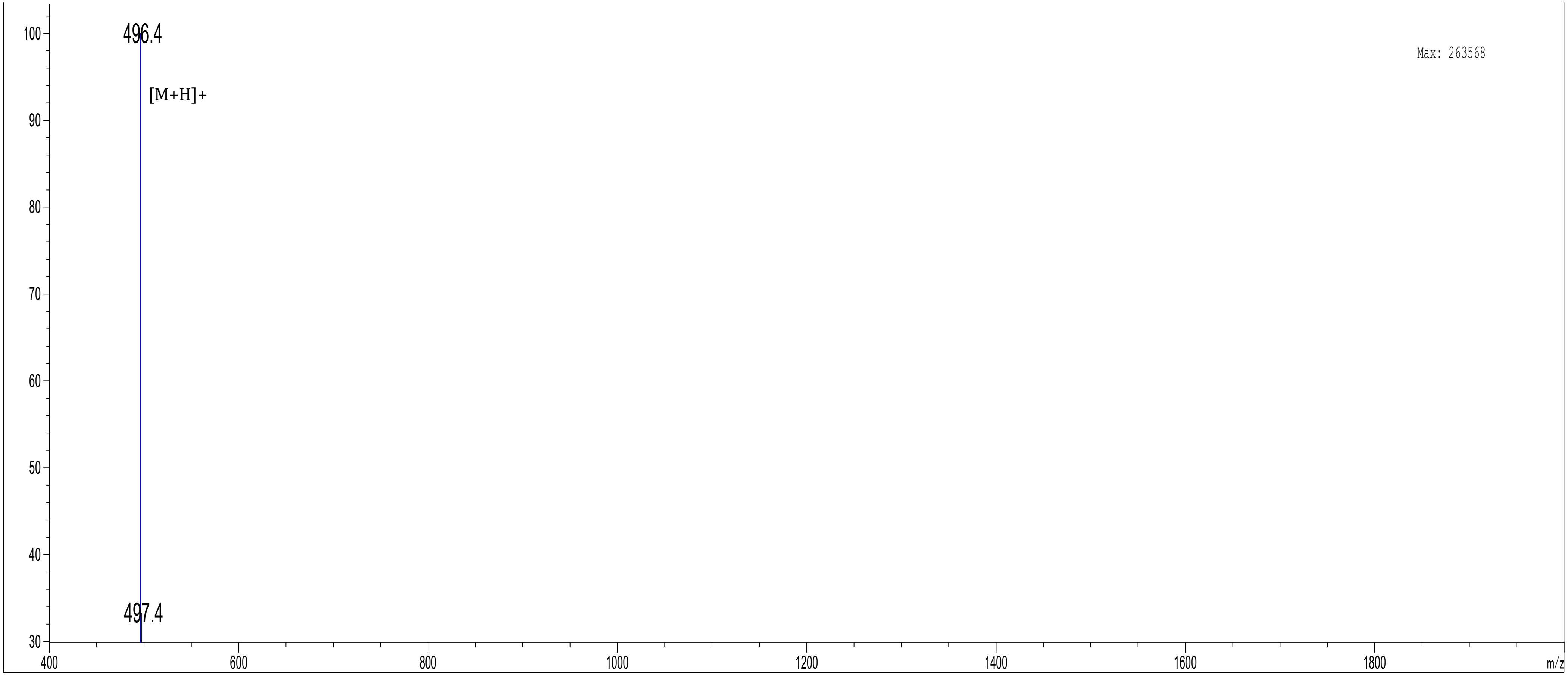

Sample Description		Instrument	Agilent-6125B		
Analyzed date:	2025-12-01	Probe:	ESI	Probe Bias:	＋4.5kv
Analyst:	Apeptide	Nebulizer Gas Flow:	1.5L/min	Detector:	1.5kv
Sample:	KYW	CDL:	-20.0v	T. Flow:	0.2ml/min
M.W.:	495.57	CDL Temp.:	250 ℃	B. Conc.:	50%H2O/50%ACN
Lot. No.:	Apep253307	Block Temp.:	200 ℃		

HPLC REPORT

Sample:	KYW	Analyzed Date:	2025-12-01
Analyst:	Apeptide		
Column:	Boston Green 0DS-AQ, 4.6*250mm, 5μm		
Solvent A	A: 0.1% Trifluoroacetic Acid in 100% Acetonitrile		
Solvent B	B: 0.1% Trifluoroacetic Acid in 100% Water		

Gradient :

	A	B
0.0min	15%	85%
25.0min	40%	60%
25.01min	100%	0%
	Stop	

Volume:	10μl
Wavelength:	220nm
Flow rate:	1.0ml/min

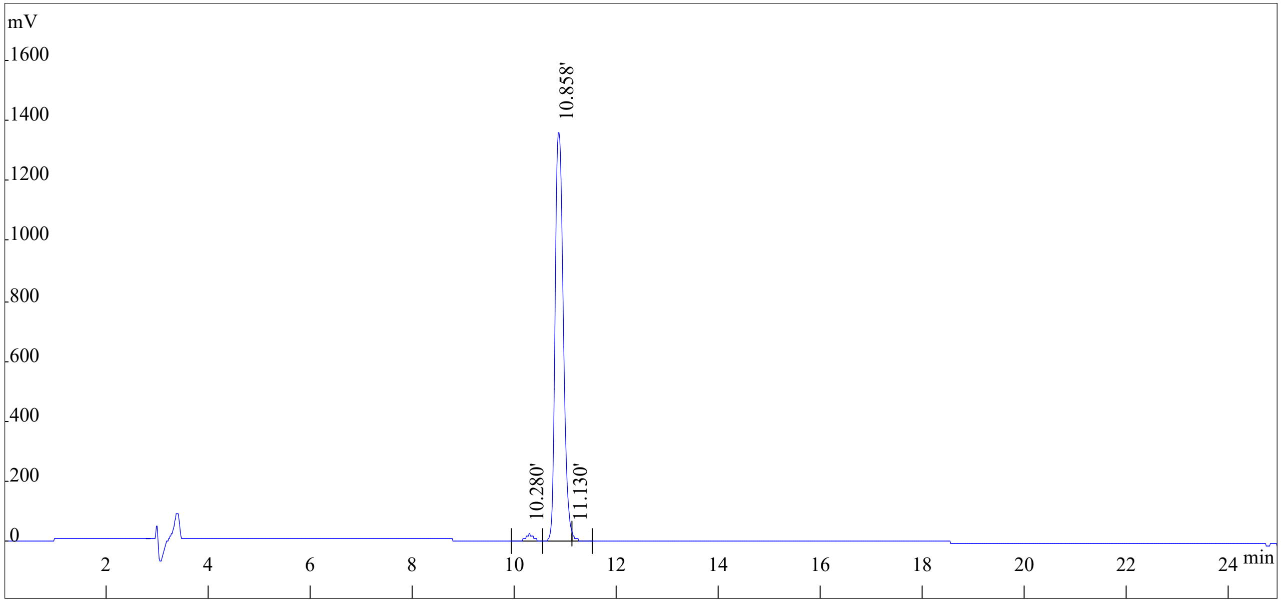

Rank	Time	Conc.	Area	Height
1	10.280	1.294	185412	19384
2	10.858	98.15	14062653	1352209
3	11.130	0.5565	79739	20451
Total		100.0000		

MASS SPECTROMETRY REPORT

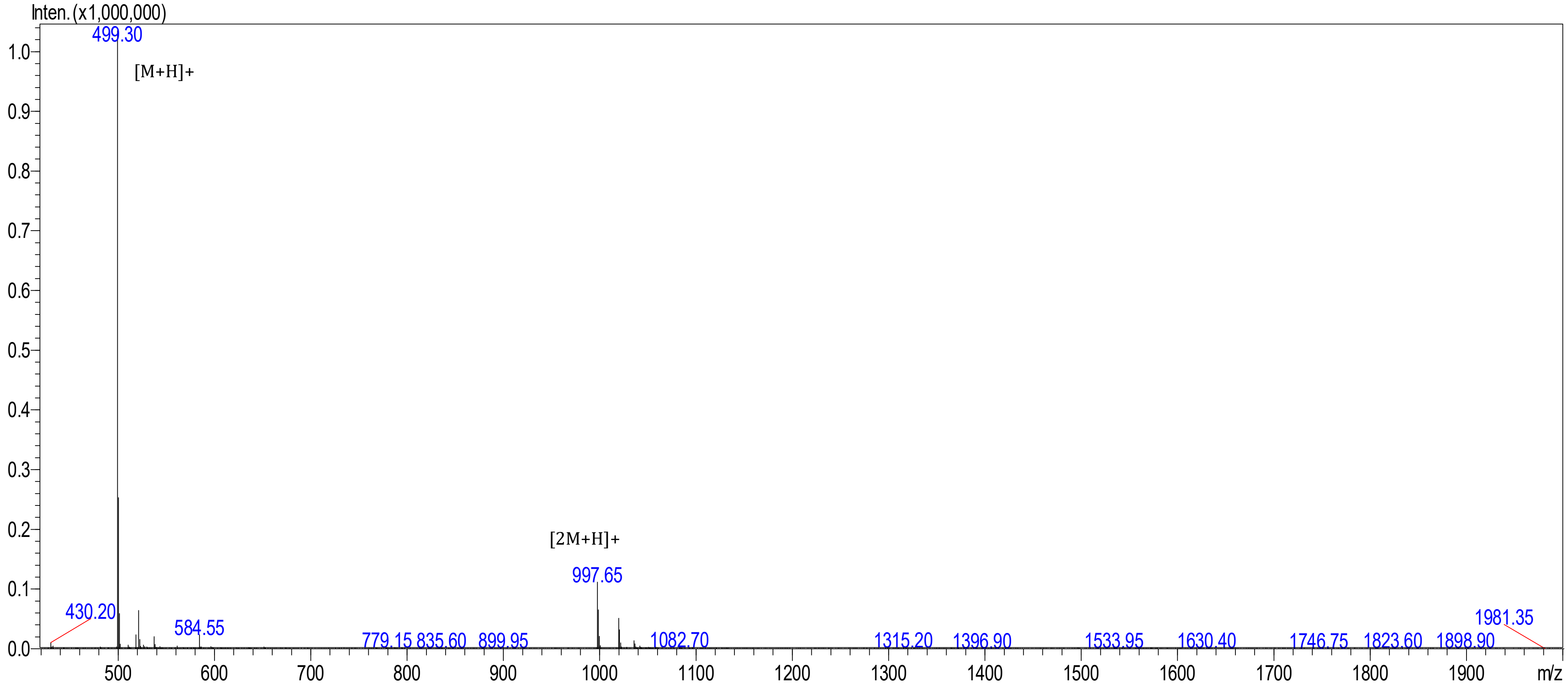

Sample Description		Instrument	SHIMADZU LCMS-2020		
Analyzed date:	2025-12-01	Probe:	ESI	Probe Bias:	+4.5kv
Analyst:	Apeptide	Nebulizer Gas Flow:	1.5L/min	Detector:	1.5kv
Sample:	PVHF	CDL:	-20.0v	T. Flow:	0.2ml/min
M.W.:	498.58	CDL Temp.:	250 ºC	B. Conc.:	50%H2O/50%ACN
Lot. No.:	Apep1308534	Block Temp.:	200 ºC		

HPLC REPORT

Sample: PVHF   Analyzed Date: 2025-12-01
Analyst: Apeptide
Column: Boston Green 0DS-AQ, 4.6*250mm, 5μm
Solvent A A: 0.1% Trifluoroacetic Acid in 100% Acetonitrile
Solvent B B: 0.1% Trifluoroacetic Acid in 100% Water
Gradient :

	A	B
0.0min	15%	85%
25.0min	40%	60%
25.01min	100%	0%
	Stop	

Volume: 10μl
Wavelength: 220nm
Flow rate: 1.0ml/min

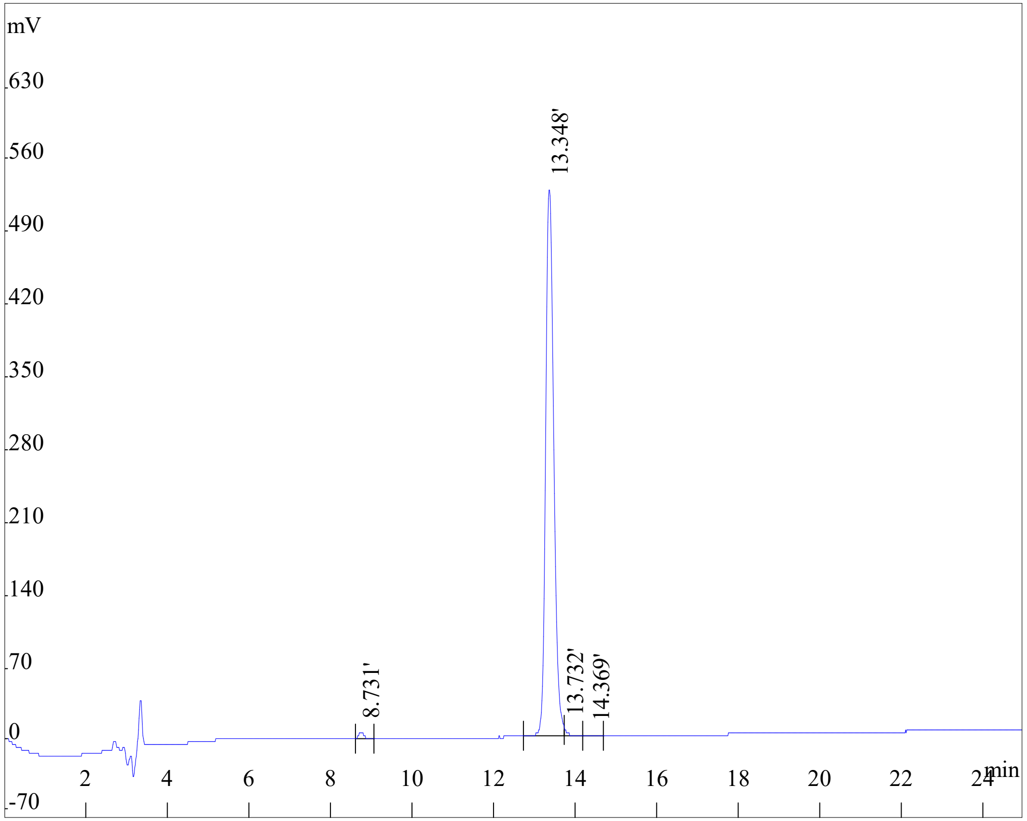

Rank	Time	Conc.	Area	Height
1	8.731	0.9037	63570	7368
2	13.348	98.35	6917630	526496
3	13.732	0.5031	35388	6592
4	14.369	0.2469	17366	1755
Total		100.0000		

HPLC REPORT

Sample:	VPVHF	Analyzed date:	2025-12-01
Analyst:	Apeptide		
Column:	Symmetrix ODS-R, 4.6*250mm, 5µm		
Solvent A	A: 0.1% Trifluoroacetic Acid in 100% Acetonitrile		
Solvent B	B: 0.1% Trifluoroacetic Acid in 100% Water		

	A	B
0.0min	16%	84%
25.0min	41%	59%
25.1min	100%	0%
30.0min	Stop	

Volume:	20µl
Wavelength:	220nm
Flow rate:	1.0ml/min

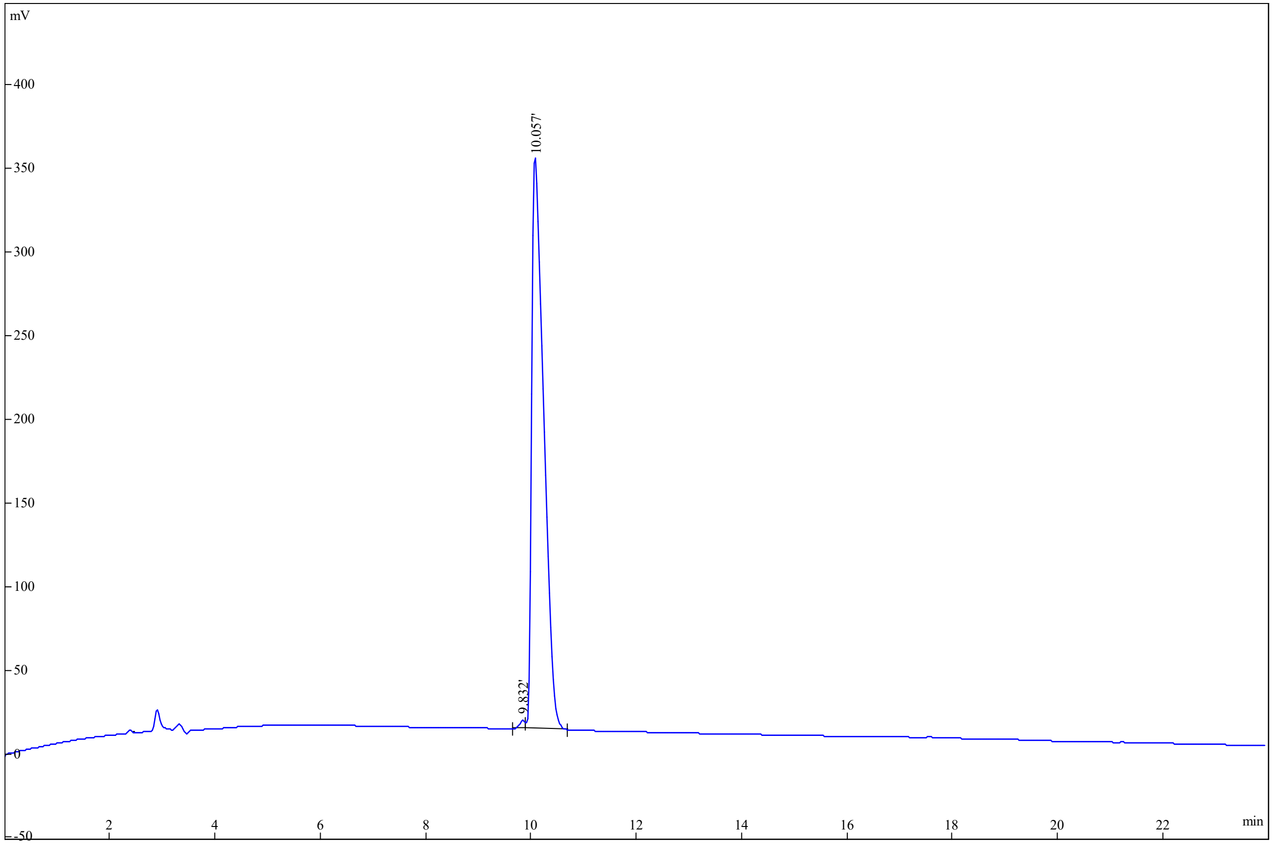

Rank	Time	Conc.	Area	Height
1	9.832	0.7265	39568	5020
2	10.057	99.28	5406936	344347
Total		100	5446504	349367

MASS SPECTROMETRY REPORT

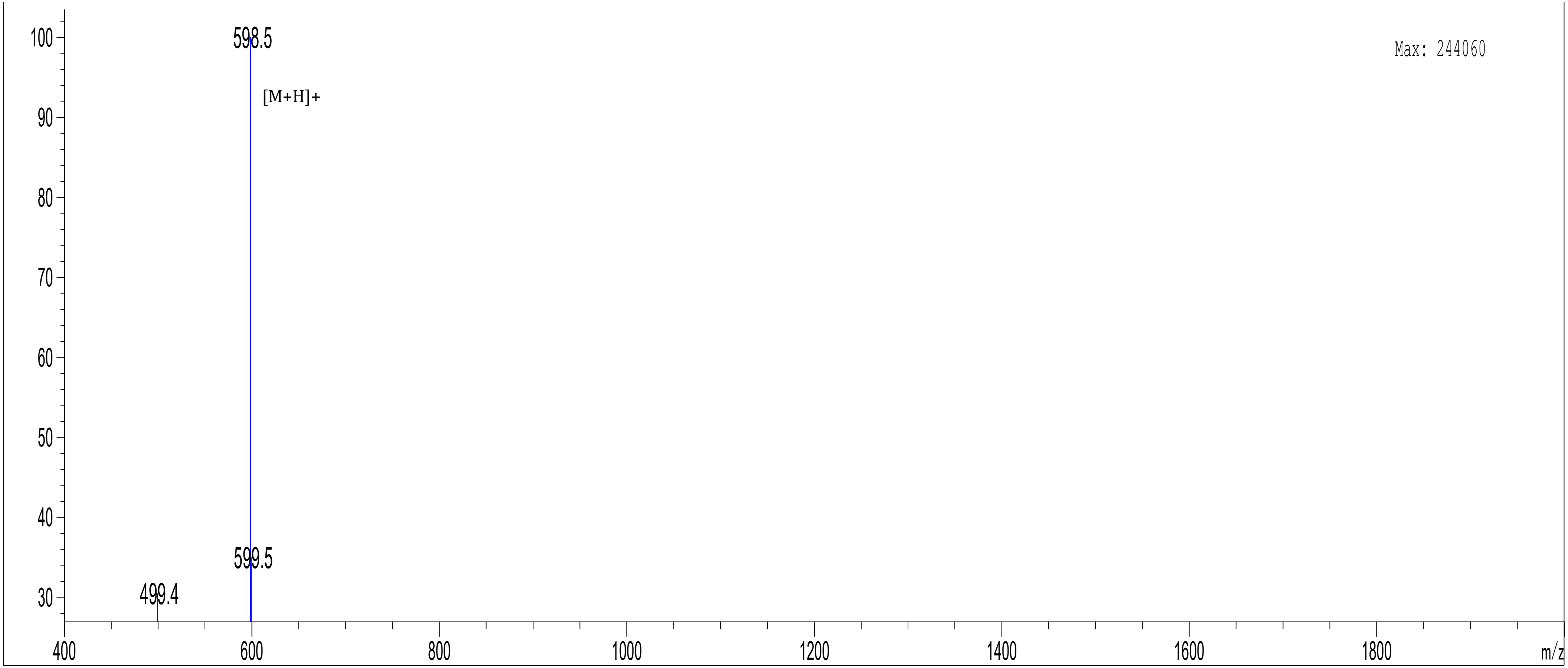

Sample Description		Instrument	Agilent-6125B		
Analyzed date:	2025-12-01	Probe:	ESI	Probe Bias:	＋4.5kv
Analyst:	Apeptide	Nebulizer Gas Flow:	1.5L/min	Detector:	1.5kv
Sample:	VPVHF	CDL:	-20.0v	T. Flow:	0.2ml/min
M.W.:	597.71	CDL Temp.:	250 °C	B. Conc.:	50%H2O/50%ACN
Lot. No.:	Apep1308532	Block Temp.:	200 °C		

HPLC REPORT

Sample: VPYPR   Analyzed date: 2025-12-01

Analyst: Apeptide

Column: Symmetrix ODS-R, 4.6*250mm, 5μm

Solvent A A: 0.1% Trifluoroacetic Acid in 100% Acetonitrile

Solvent B B: 0.1% Trifluoroacetic Acid in 100% Water

	A	B
0.0min	12%	88%
25.0min	37%	63%
25.1min	100%	0%
30.0min	Stop	

Volume: 20μl

Wavelength: 220nm

Flow rate: 1.0ml/min

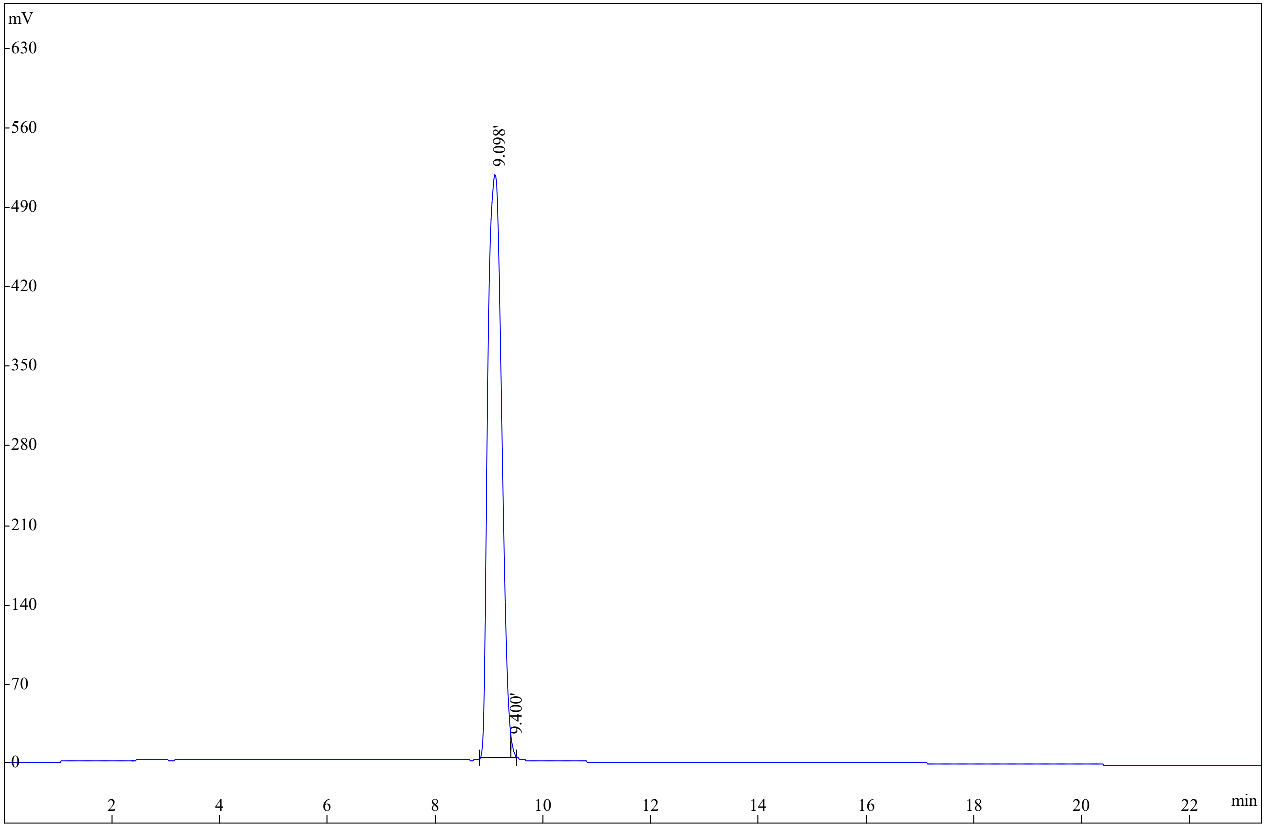

Rank	Time	Conc.	Area	Height
1	9.098	99.6193	8598304	513558
2	9.400	0.3807	32857	13892
Total		100	8631161	527450

MASS SPECTROMETRY REPORT

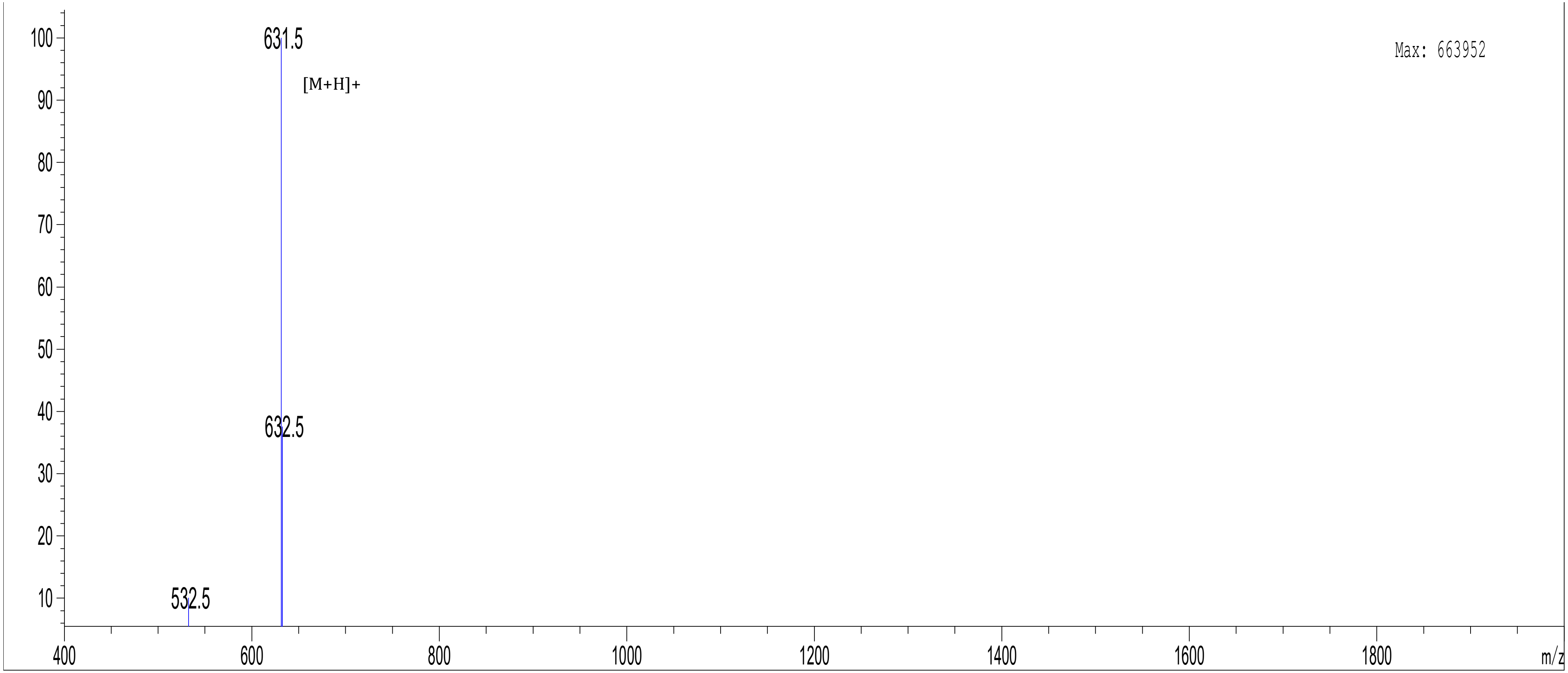

Sample Description		Instrument	Agilent-6125B		
Analyzed date:	2025-12-01	Probe:	ESI	Probe Bias:	＋4.5kv
Analyst:	Apeptide	Nebulizer Gas Flow:	1.5L/min	Detector:	1.5kv
Sample:	VPYPR	CDL:	-20.0v	T. Flow:	0.2ml/min
M.W.:	630.74	CDL Temp.:	250 °C	B. Conc.:	50%H2O/50%ACN
Lot. No.:	Apep1308531	Block Temp.:	200 °C		
