# Supplementary material for: Identification of Novel AChE-Targeting Neuroprotective Peptides from Pacific Oyster (Crassostrea gigas): An Integrated Pipeline of Peptidomics, Molecular Dynamics, and Cellular Validation
Source: Mar Drugs. 2026 Aug 25;24(9):298. doi: 10.3390/md24090298 (PMC13608502; doi:10.3390/md24090298)

MS of HHTF:

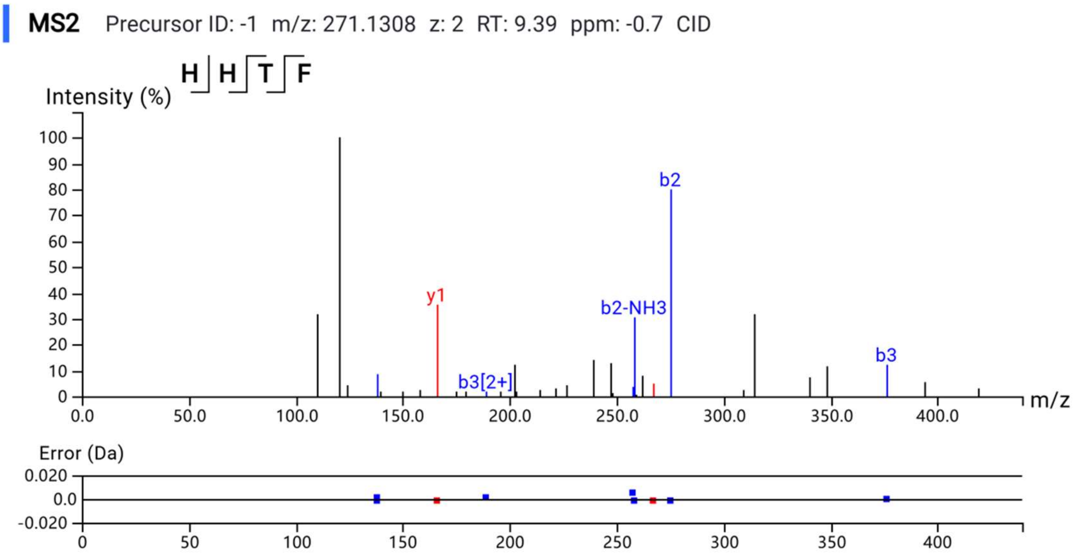

MS2 Precursor ID: -1 m/z: 271.1308 z: 2 RT: 9.39 ppm: -0.7 CID
H H T F
Intensity (%)
b2
y1
b2-NH3
b3
b3[2+]
m/z
Error (Da)

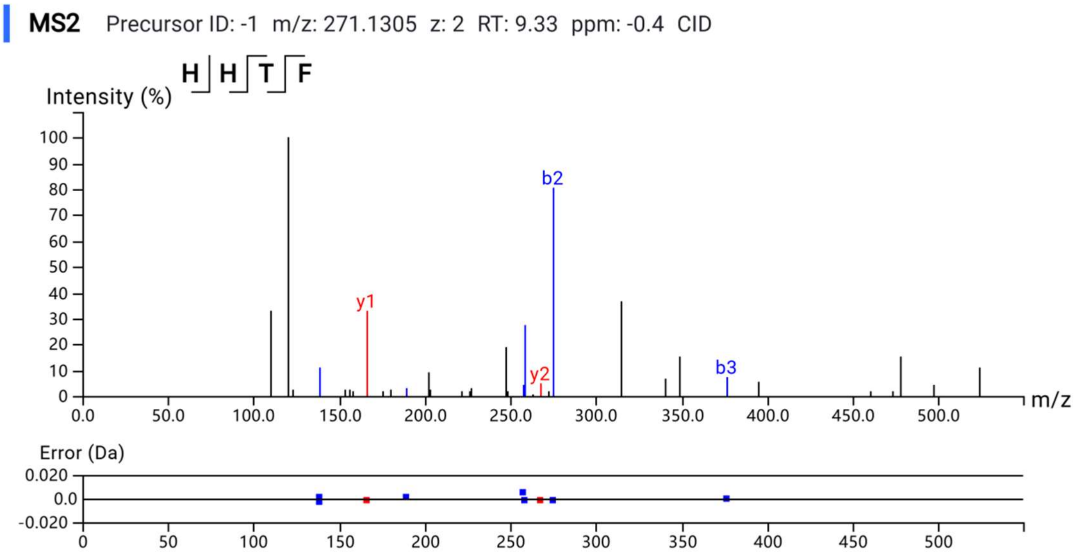

MS2 Precursor ID: -1 m/z: 271.1305 z: 2 RT: 9.33 ppm: -0.4 CID
H H T F
Intensity (%)
b2
y1
y2
b3
m/z
Error (Da)

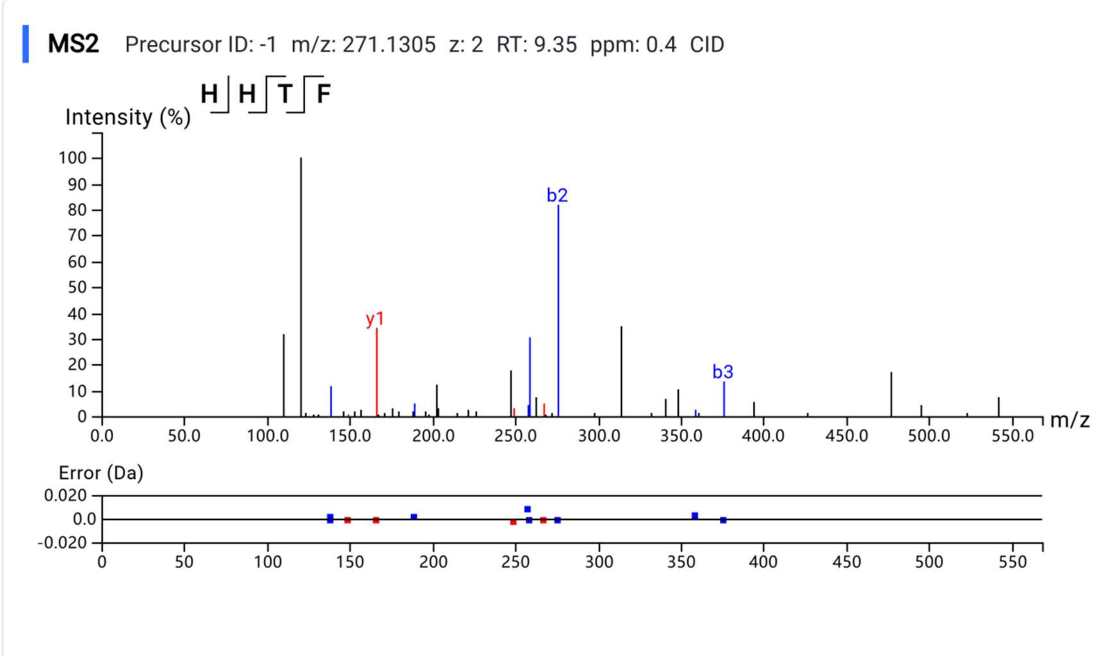
MS2 Precursor ID: -1 m/z: 271.1305 z: 2 RT: 9.35 ppm: 0.4 CID
H H T F
Intensity (%)
y1
b2
b3
m/z
Error (Da)

MS of VPVHF:

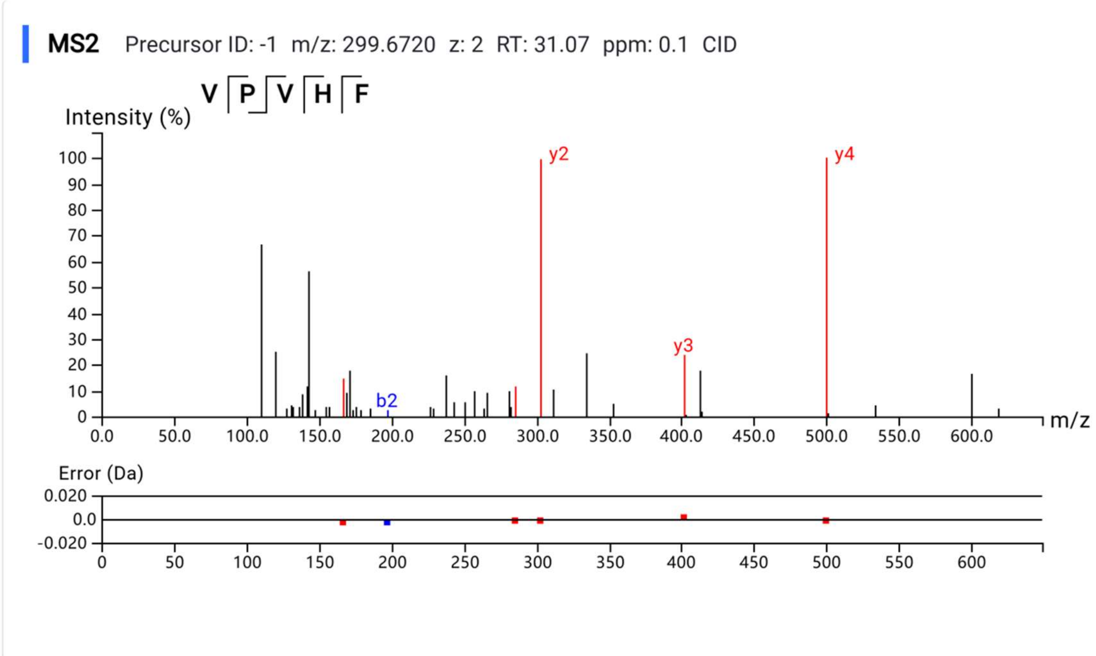
MS2 Precursor ID: -1 m/z: 299.6720 z: 2 RT: 31.07 ppm: 0.1 CID
V P V H F
Intensity (%)
y2
y4
y3
b2
m/z
Error (Da)

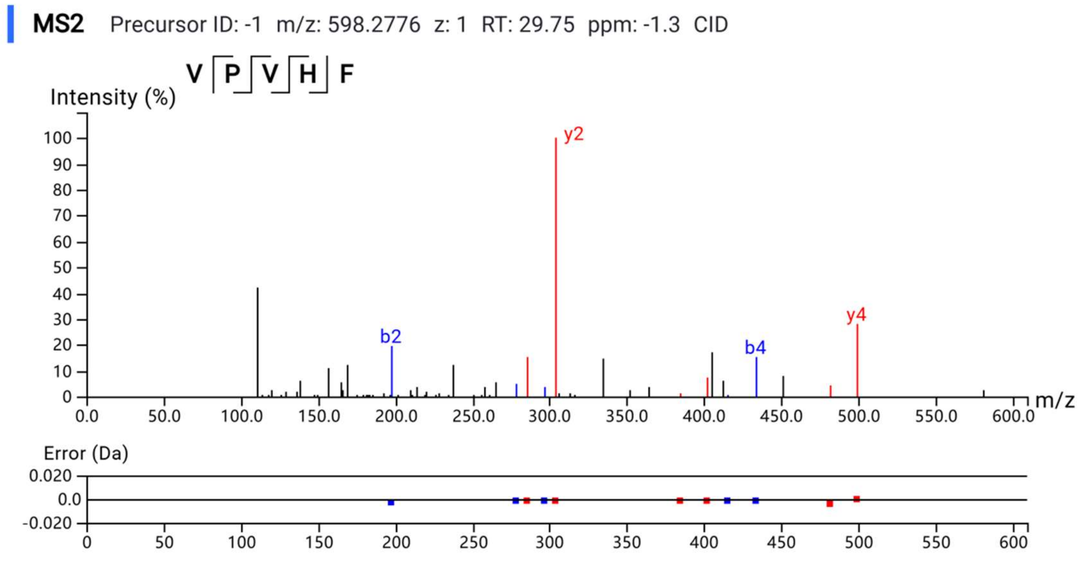
MS2 Precursor ID: -1 m/z: 598.2776 z: 1 RT: 29.75 ppm: -1.3 CID
V P V H F
Intensity (%)
y2
y4
b2
b4
m/z
Error (Da)

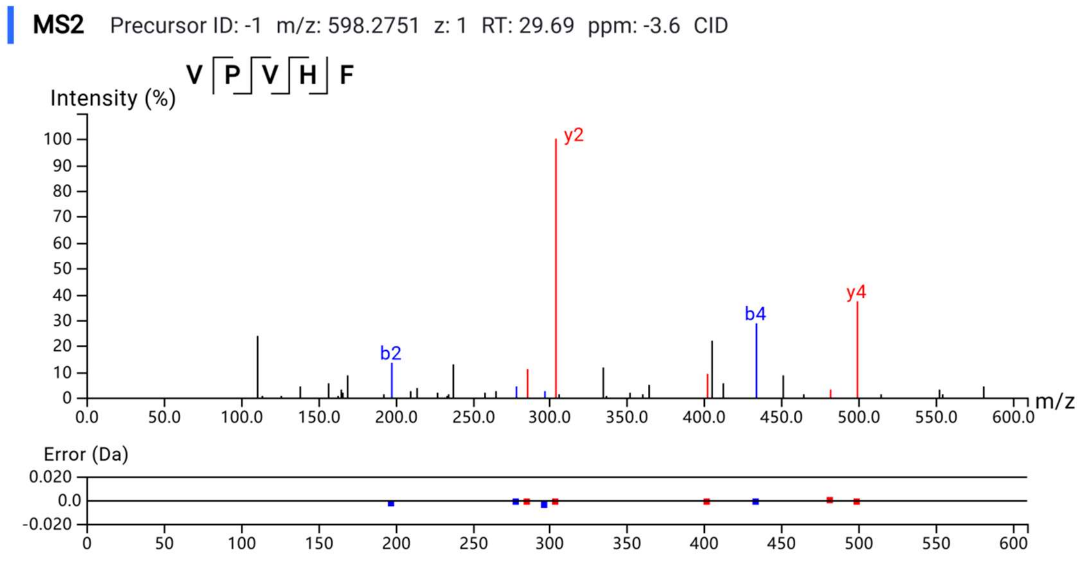
MS2 Precursor ID: -1 m/z: 598.2751 z: 1 RT: 29.69 ppm: -3.6 CID
V P V H F
Intensity (%)
y2
y4
b2
b4
m/z
Error (Da)

MS of VPVHF:

MS2 Precursor ID: -1 m/z: 499.2685 z: 1 RT: 29.56 ppm: 1.0 CID

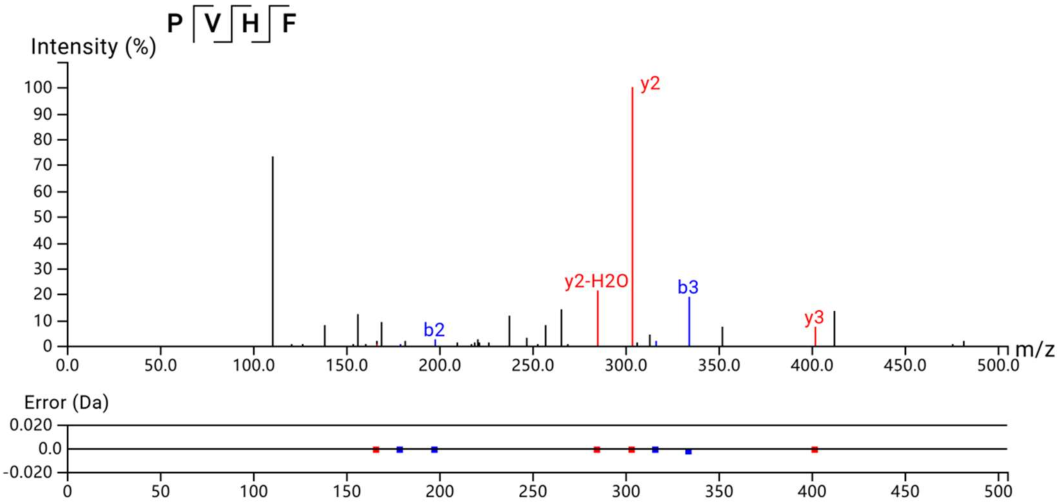

MS2 Precursor ID: -1 m/z: 498.7312 z: 1 RT: 29.41 ppm: -1.9 CID

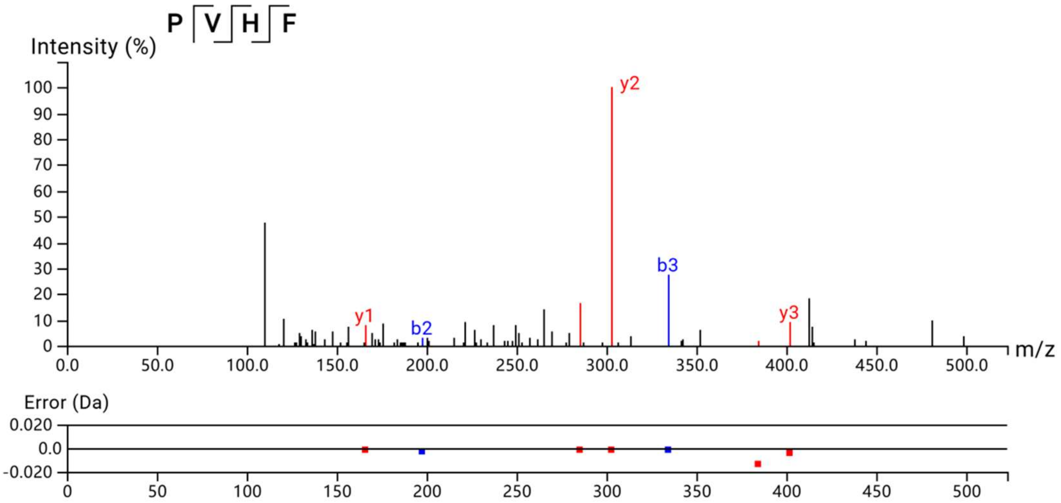

MS2 Precursor ID: -1 m/z: 499.2699 z: 1 RT: 29.47 ppm: 1.8 CID

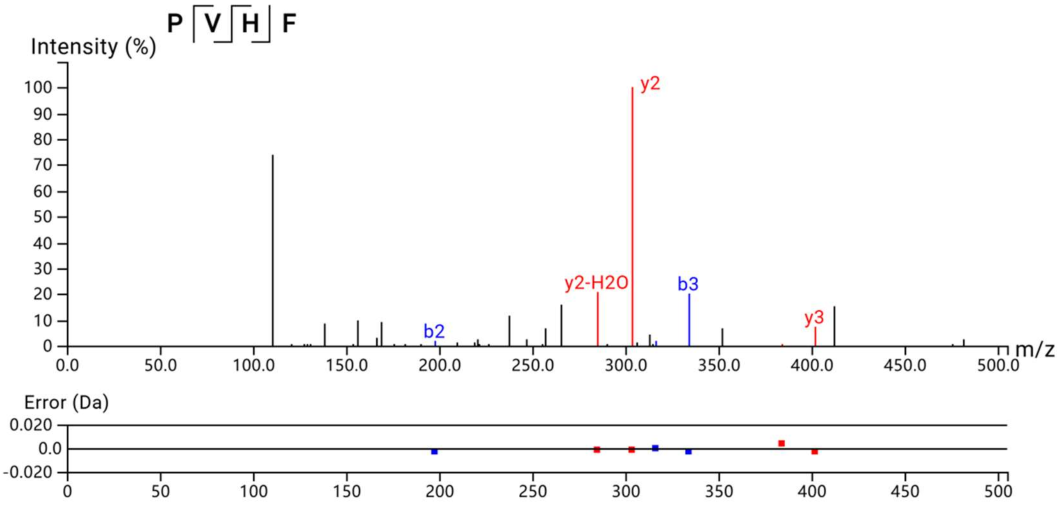

Supplement: Supplementary file 1 [file marinedrugs-24-00298-s001.zip › MS of three peptides.pdf]
